# Duration-dependent impact of cardiometabolic diseases and multimorbidity on all-cause and cause-specific mortality: a prospective cohort study of 0.5 million participants

**DOI:** 10.1186/s12933-023-01858-9

**Published:** 2023-06-12

**Authors:** Yuting Han, Yizhen Hu, Canqing Yu, Dianjianyi Sun, Yuanjie Pang, Pei Pei, Ling Yang, Yiping Chen, Huaidong Du, Jingchao Liu, Dan Schmidt, Daniel Avery, Junshi Chen, Zhengming Chen, Liming Li, Jun Lv

**Affiliations:** 1 Department of Epidemiology & Biostatistics, School of Public Health, Peking University, Beijing, China; 2 Peking University Center for Public Health and Epidemic Preparedness & Response, Beijing, China; 3 Key Laboratory of Epidemiology of Major Diseases (Peking University), Ministry of Education, Beijing, China; 4 Medical Research Council Population Health Research Unit at the University of Oxford, Oxford, UK; 5 Clinical Trial Service Unit & Epidemiological Studies Unit (CTSU), Nuffield Department of Population Health, University of Oxford, Oxford, UK; 6 NCDs Prevention and Control Department, Wuzhong CDC, Suzhou, Jiangsu China; 7 China National Center for Food Safety Risk Assessment, Beijing, China

**Keywords:** Cardiometabolic disease, Multimorbidity, Mortality, Prospective cohort

## Abstract

**Background:**

The association of incident cardiometabolic multimorbidity (CMM) with mortality risk is rarely studied, and neither are the durations of cardiometabolic diseases (CMDs). Whether the association patterns of CMD durations with mortality change as individuals progress from one CMD to CMM is unclear.

**Methods:**

Data from China Kadoorie Biobank of 512,720 participants aged 30–79 was used. CMM was defined as the simultaneous presence of two or more CMDs of interest, including diabetes, ischemic heart disease, and stroke. Cox regression was used to estimate the hazard ratios (HRs) and 95% confidence intervals (CIs) for the duration-dependent associations of CMDs and CMM with all-cause and cause-specific mortality. All information on exposures of interest was updated during follow-up.

**Results:**

During a median follow-up of 12.1 years, 99,770 participants experienced at least one incident CMD, and 56,549 deaths were documented. Among 463,178 participants free of three CMDs at baseline, compared with no CMD during follow-up, the adjusted HRs (95% CIs) between CMM and all-cause mortality, mortality from circulatory system diseases, respiratory system diseases, cancer, and other causes were 2.93 (2.80–3.07), 5.05 (4.74–5.37), 2.72 (2.35–3.14), 1.30 (1.16–1.45), and 2.30 (2.02–2.61), respectively. All CMDs exhibited a high mortality risk in the first year of diagnosis. Subsequently, with prolonged disease duration, mortality risk increased for diabetes, decreased for IHD, and sustained at a high level for stroke. With the presence of CMM, the above association estimates inflated, but the pattern of which remained.

**Conclusion:**

Among Chinese adults, mortality risk increased with the number of the CMDs and changed with prolonged disease duration, the patterns of which varied among the three CMDs.

**Supplementary Information:**

The online version contains supplementary material available at 10.1186/s12933-023-01858-9.

## Introduction

Due to rapid urbanization, globalization of unhealthy lifestyles, and population aging, cardiometabolic diseases (CMDs), including diabetes, heart attack, and stroke, have become one of the major public health problems and caused around half of the non-communicable diseases (NCDs) deaths and a quarter of disability-adjusted life years of NCDs in 2019 [1]. Also, more individuals develop and live with cardiometabolic multimorbidity (CMM), one of the most replicable multimorbidity patterns among different study populations, study designs, and statistical methods [2].

Previous studies, including ours, showed that CMM significantly increased mortality risk [3–13]. However, most previous cohort studies examined the association between baseline disease status and mortality risk without considering changes in disease status during follow-up [3, 5–10, 12]. Only using baseline prevalent cases may bias the association estimates because fatal cases and cases with progressive conditions were hardly enrolled in the cohorts. The varied disease duration of the prevalent cases may impact results. Several studies observed that the risk of mortality changed with the duration of diabetes, though the conclusion remains controversial [11, 13–21]. Similar studies for ischemic heart disease (IHD) and stroke are limited [14, 15, 18].

There are still some unknowns about the prognosis of CMDs and CMM. For example, do the associations between the CMD duration and risks of different cause-specific mortality exhibit the same pattern? How does mortality risk change as individuals progress from one CMD to CMM? Since the quantity and quality of healthcare resources influence the prognosis of CMDs [22], filling the evidence gaps in the Chinese population, in which the medical resources are limited and imbalanced distributed between urban and rural areas [23], will benefit the management of CMD.

This study used data from the China Kadoorie Biobank (CKB) of 0.5 million Chinese adults. We aimed to examine the associations of time-updated CMDs and CMM, specifically expressed as the number and combination of CMDs, with the risks of all-cause and cause-specific mortality. We also explored how the risk of mortality was jointly influenced by the duration of CMDs and the presence of CMM. In line with previous studies [3, 5, 6, 24, 25], the CMDs of interest included diabetes, IHD, and stroke, and the CMM was defined as the simultaneous presence of two or more CMDs.

## Methods

### Study design and population

Details of the study design and implementation of the CKB have been reported previously [26]. Briefly, the CKB is a large prospective cohort recruiting 512,723 participants aged 30–79 from five urban and five rural areas. The baseline survey took place during 2004–2008. All participants provided written informed consent and completed interviewer-administered laptop-based questionnaires, which collected information on socioeconomic characteristics, lifestyle (e.g., smoking, alcohol drinking, dietary habits, and physical activity), and personal and family medical history. Physical measurements (e.g., weight, height, and blood pressure) were taken using well-calibrated instruments and following standard procedures. Details of the baseline data are available online [27]. All participants were followed up through the linkage to disease and mortality registries and national insurance claim database to obtain updated disease and vital status and the diagnosis and death dates. All events were coded according to the International Classification of Diseases, 10th Revision (ICD-10) by trained staff blinded to baseline information.

### Assessment of cardiometabolic diseases and multimorbidity

At baseline, participants were asked: “Has a doctor ever told you that you had the following diseases?”, followed by a list of common conditions, including diabetes, IHD, and stroke (including transient ischemic attack). Participants who answered “yes” were further asked about their age at first diagnosis and current use of medications, including aspirin, statins, and medications to lower blood pressure for IHD and stroke cases, and insulin and metformin for diabetes cases. Besides, an on-site random plasma glucose (RPG) testing was conducted using the SureStep Plus system (Lifespan) with a record of fasting time. Participants without a previously diagnosed diabetes plus an RPG between 140 and 200 mg/dL (7.8–11.0 mmol/L) and a fasting time of < 8 h were invited for a fasting plasma glucose (FPG) testing the following day. Screened diabetes was defined as: (1) an RPG of ≥ 126 mg/dL (≥ 7.0 mmol/L) with a fasting time of ≥ 8 h; or (2) an RPG of ≥ 200 mg/dL (≥ 11.1 mmol/L) with a fasting time < 8 h; or (3) an FPG of 126 mg/dL (≥ 7.0 mmol/L) on subsequent testing. Prevalent diabetes at baseline included both previously diagnosed and screened diabetes.

In addition to baseline assessment, the status of CMDs and CMM were updated during the follow-up, i.e., participants with incident CMD were considered exposed from the date of diagnosis. New cases were identified by the ICD-10 code: diabetes (E10–E14), IHD (I20–I25), and stroke (I60, I61, I63, and I64). Considering that the CMD newly documented within 30 days before death is likely to be the cause of death, we did not consider it a change in the status of CMDs.

The status of CMDs was expressed by the number of CMDs (0, 1, or ≥ 2; with ≥ 2 as CMM) or the combination of CMDs that included eight mutually exclusive groups: (1) without any CMDs; (2) diabetes; (3) IHD; (4) stroke; (5) diabetes and IHD; (6) diabetes and stroke; (7) IHD and stroke; or (8) diabetes, IHD, and stroke. The time-updated duration of a certain CMD was defined as the time interval between the first diagnosis of the CMD and a time point during the follow-up. The duration (years) was categorized into nine groups: without a certain CMD, 0-, < 1, 1-, 5-, 10-, 15-, 20-, 25-, and 30-.

### Ascertainment of mortality outcomes

Causes of death were ascertained chiefly by death certificates and supplemented by reviews of medical records and verbal autopsies using validated instruments. The main outcomes of this study were all-cause mortality and cause-specific mortality from circulatory system diseases (ICD-10: I00-I99), respiratory system diseases (J00–J99), cancer (C00–C97), and all other causes.

### Statistical methods

Three participants with missing baseline data for body mass index (BMI) or age at first diagnosis of CMDs were first excluded from the analyses, leaving 512,720 participants in the study. We further excluded participants who had diabetes, IHD, or stroke at baseline, with 463,178 participants remaining in some analyses. Participants were considered at risk from enrollment to death, loss to follow-up, or Dec 31, 2018, whichever came first.

Baseline characteristics of participants were described according to the CMD status at the end of 2018, with adjustment for age, sex, and study area. The Cox proportional hazards models estimated the hazard ratios (HRs) and 95% confidence intervals (CIs) for all associations of interest. The models were stratified by age in the 5-year interval and study area and used age as the time scale. Covariates included sex, education, household income, marital status, family history of diabetes, heart attack, or stroke, smoking, alcohol drinking, dietary habits, physical activity, BMI, waist circumference, prevalent hypertension, kidney diseases, and rheumatoid heart disease.

To avoid the potential survivor bias, we assessed the associations of time-updated CMDs and CMM (by number or combination of CMDs separately) during follow-up with all-cause and cause-specific mortality in 463,178 participants who were free from diabetes, IHD, and stroke at baseline.

We further assessed the association of time-updated duration of each CMD with all-cause and cause-specific mortality. Because the longest follow-up for the current data was less than 15 years, we included participants who had diabetes, IHD, or stroke at baseline, with a total of 512,720 participants in this part of analysis. The baseline CMD patients can additionally help us understand the mortality risk associated with disease duration longer than 15 years. To explore how the risk of mortality was jointly influenced by the duration of CMDs and the presence of CMM, the models included the main effect of the duration of certain CMD and an interaction term between the duration of the CMD and presence of the other two CMDs (either or both).

We performed several sensitivity analyses to test how the results depended on the study population, disease definition, and confounding control. First, in the association analyses of CMDs and CMM with mortality, we used all 512,720 participants and employed another two strategies to define disease status: (1) according to disease status at baseline; and (2) according to disease status at baseline and during follow-up. Second, since the case fatality rate of hemorrhagic stroke (HS) is greatly higher than that of ischemic stroke (IS), we repeated the analyses of updated CMDs and CMM during follow-up with mortality in 463,178 participants by: (1) including IS (I63) and HS (I61) as separate diseases and forming 16 mutually exclusive combination groups; (2) replacing stroke with IS. Other sensitivity analyses included excluding the deaths occurring in the first 2-year of follow-up and additionally adjusting for the use of antihypertensive medications, statins, and aspirin, and prevalent cancer, emphysema, and bronchitis at baseline.

Stratified analyses were conducted for associations between the duration of CMDs and mortality. The interactions were tested by using the likelihood ratio test comparing models with and without a cross-product term.

We performed all statistical analyses using Stata (version 17, StataCorp) and plotted forest plots using R (version 4.0.3). Two-tailed *P* < 0.05 indicated statistical significance.

## Results

### Description of study population

Of the 512,720 participants, the mean baseline age was 52.0 ± 10.7 years, and 41.0% were males (Table 1). During a median follow-up of 12.1 years (interquartile range, 11.1–13.1; total person-years, 6,008,683), 99,770 participants experienced at least one incident CMD. At the end of 2018, 102,003 (19.9%) participants had one CMD [49,362 had diabetes, 58,489 had IHD, and 63,777 had stroke (Additional file 1: Table S1)] and 32,546 (6.3%) had CMM. Compared with participants without or with only one CMD, participants with CMM were more likely to be older and urban residents, and have unhealthy lifestyles, family history of CMDs, and prevalent hypertension at baseline. During the follow-up, 56,549 deaths were documented, including 23,289 (41.2%) deaths from circulatory system diseases, 5362 (9.5%) from respiratory system diseases, 17,691 (31.3%) from cancer, and 10,207 (18.1%) from other causes.

**Table 1:** Baseline characteristics of participants by cardiometabolic disease status at the end of 2018

	No CMD	Diabetes	IHD	Stroke	Diabetes and IHD	Diabetes and stroke	IHD and stroke	Diabetes and IHD and stroke	Total
No. of participants at baseline	463,178	26,414	11,897	6471	2348	1186	876	350	512,720
No. of participants at the end of 2018	378,171	30,389	33,553	38,061	6830	7610	13,573	4533	512,720
Age at baseline, year (SD)	50.0 (10.2)	54.6 (9.7)	57.4 (10.0)	57.9 (9.8)	59.8 (9.0)	59.4 (8.9)	61.4 (8.9)	62.0 (8.4)	52.0 (10.7)
Female, %	58.5	62.1	63.4	53.8	68.0	59.8	61.4	65.3	59.0
Urban residents, %	41.4	49.5	51.4	45.0	61.4	55.9	63.6	70.7	44.1
Middle school and above, %	49.2	48.9	50.4	47.9	49.4	48.6	50.7	50.1	49.2
Household income ≥ 20,000 yuan/year, %	42.4	43.7	44.2	41.1	44.6	42.4	45.6	46.5	42.7
Married, %	90.3	90.7	91.1	90.7	91.7	91.5	91.9	92.4	90.6
Family history of disease, %									
Diabetes	6.0	15.7	7.0	5.7	16.1	14.9	6.7	15.9	7.0
IHD	3.0	3.6	4.5	3.4	4.6	3.6	4.3	4.5	3.3
Stroke	17.1	19.1	19.9	22.4	20.3	22.3	22.5	24.4	18.2
Any CMD	23.6	32.8	27.8	28.5	34.0	34.0	29.5	37.1	25.4
Lifestyle factors, %									
Current smoking or having quit because of illness	29.1	28.9	31.0	30.3	31.4	29.2	31.1	29.9	29.4
Excessive alcohol drinking^a^	10.4	11.5	11.3	12.4	11.8	12.7	12.3	12.4	10.8
Less healthy dietary habits^b^	97.8	98.1	97.9	98.1	98.1	98.2	98.0	98.3	97.9
Being physically inactive^c^	49.2	54.8	52.7	52.3	58.4	57.7	55.2	57.6	50.5
BMI < 18.5 kg/m^2^ or BMI ≥ 24.0 kg/m^2^	45.0	63.6	53.2	50.8	68.8	64.7	56.0	69.0	48.1
Waist circumference (M/F) ≥ 90/85 cm	20.8	41.5	28.5	26.0	47.7	43.9	31.1	48.4	24.3
Use of medications, %									
Antihypertensive medications^d^	8.4	17.1	18.3	18.9	26.9	26.8	25.1	34.2	12.2
Aspirin	0.4	1.0	2.4	2.1	3.5	2.4	3.5	4.4	1.0
Statins	0.1	0.4	0.5	0.4	1.0	0.6	0.8	1.2	0.2
Prevalent comorbidities, %									
Hypertension	30.3	46.1	40.7	49.6	55.2	59.4	51.0	64.1	35.2
Rheumatic heart disease	0.2	0.1	0.3	0.2	0.3	0.1	0.3	0.2	0.2
Kidney diseases	1.3	1.7	1.9	1.6	2.2	1.7	2.2	2.8	1.5
Cancer	0.5	0.7	0.4	0.4	0.5	0.6	0.5	0.5	0.5
Chronic bronchitis or emphysema	7.1	6.6	9.1	6.8	7.9	6.0	7.7	6.9	7.2

CMD, cardiometabolic disease; IHD, ischemic heart disease; BMI, body mass index

All percentages were adjusted for age at baseline, sex, and study areas, as appropriate

For participants who died during follow-up, the cardiometabolic disease status at the time of death was used

^a^Excessive alcohol drinking was defined as daily drinking ≥ 30 g/day of pure alcohol or having stopped regular drinking habit

^b^Less healthy dietary habit was defined as eating vegetables, fruits, and eggs less than daily, and eating red meat daily or less than weekly

^c^Being physically inactive was defined as engaging in a sex- and age-specific lower half of total physical activity

^d^Antihypertensive medications included ACE inhibitors, beta-blockers, diuretics, and calcium channel blockers

### CMDs, CMM, and mortality

**Fig. 1:**
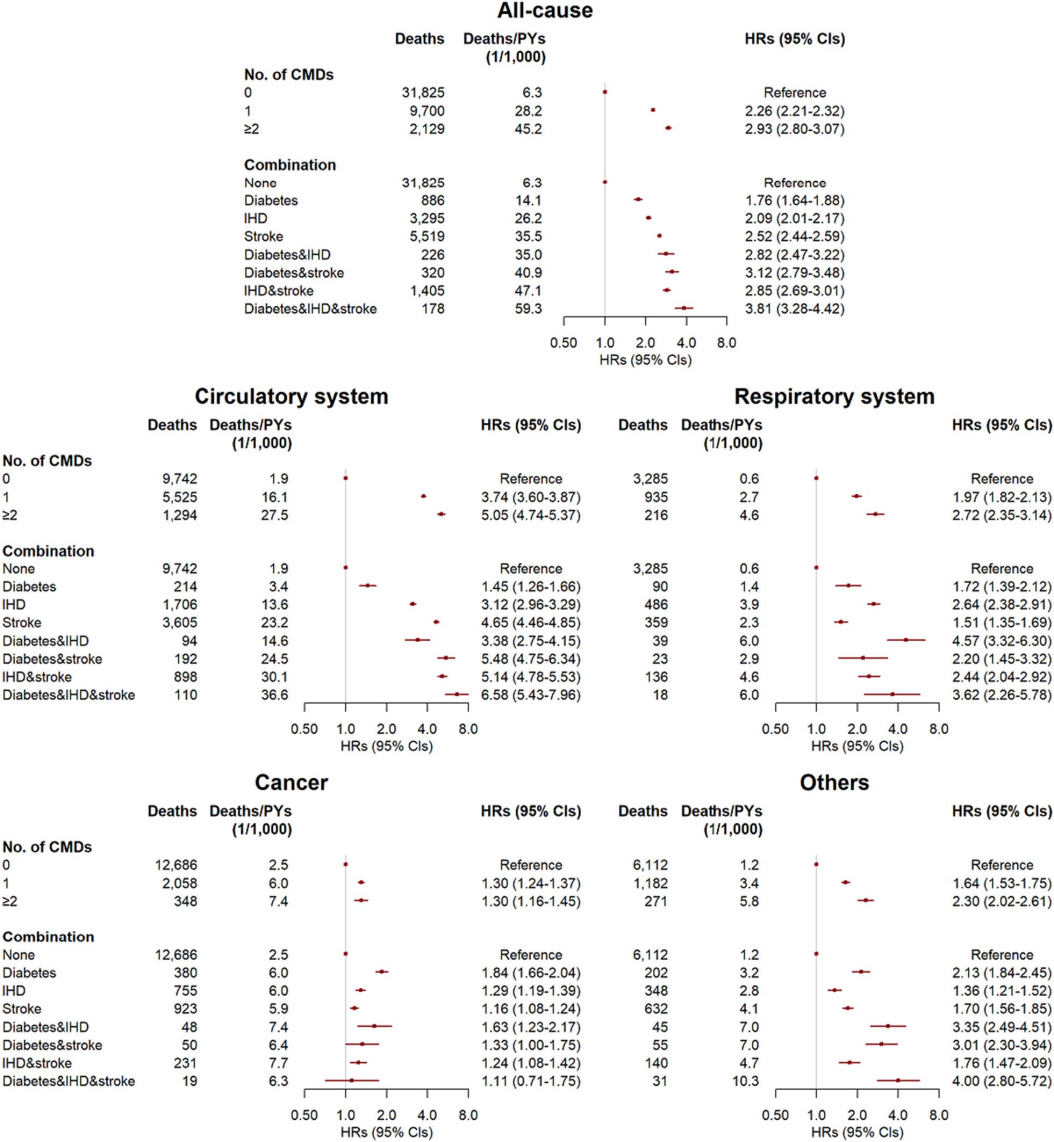
Risks of all-cause and cause-specific mortality by updated cardiometabolic disease status during follow-up in 463,178 participants. PY, person-year; HR, hazard ratio; CI, confidence interval; CMD, cardiometabolic disease; IHD, ischemic heart disease. CMDs included diabetes, IHD, and stroke. Participants with a prior diagnosis of diabetes, IHD, or stroke at baseline were excluded. Multivariable models were stratified by age in the 5-year interval and study area, and adjusted for sex, education, household income, marital status, family history of diabetes, heart attack or stroke, smoking, alcohol drinking, dietary habits, physical activity, body mass index, waist circumference, prevalent hypertension, kidney diseases, and rheumatic heart disease

In the analysis of 463,178 free of CMDs at baseline, compared with participants without any new onset of CMDs during follow-up, the multivariable-adjusted HRs (95% CIs) of all-cause mortality for participants who developed one CMD and CMM were 2.26 (2.21–2.32) and 2.93 (2.80–3.07), respectively (Fig. 1). The corresponding HRs (95% CIs) of cause-specific mortality for those with CMM were 5.05 (4.74–5.37) for circulatory system diseases, 2.72 (2.35–3.14) for respiratory diseases, 1.30 (1.16–1.45) for cancer, and 2.30 (2.02–2.61) for other causes. Also, compared with those without any CMD, the HRs (CIs) of all-cause mortality were 1.76 (1.64–1.88), 2.09 (2.01–2.17), and 2.52 (2.44–2.59) for those only with diabetes, IHD, or stroke, respectively, and 2.82 (2.47–3.22) for those with diabetes and IHD, 3.12 (2.79–3.48) for those with diabetes and stroke, 2.85 (2.69–3.01) for those with IHD and stroke, and 3.81 (3.28–4.42) for those with three CMDs.

The pattern of associations of CMDs and CMM with mortality from circulatory system diseases was similar to that with all-cause mortality, but the former had larger association estimates (Fig. 1). The relative risks of mortality from circulatory system diseases were higher for participants with stroke, either presented individually or in the form of CMM. Similar links were also observed between IHD and mortality from respiratory system diseases and between diabetes and mortality from cancer or other causes.

In the sensitivity analysis of all 512,720 participants and employing another two strategies to define disease status, for IHD and stroke, the strength of the association of mortality with time-updated disease status during follow-up was stronger than that with baseline disease status. In contrast, the opposite was observed for diabetes (Fig. 1, Additional file 1: Figure S1). When the CMD status was defined according to both information at baseline and during follow-up, the results were broadly similar to that in the primary analysis (Additional file 1: Figure S2).

### CMD duration and mortality

**Fig. 2:**
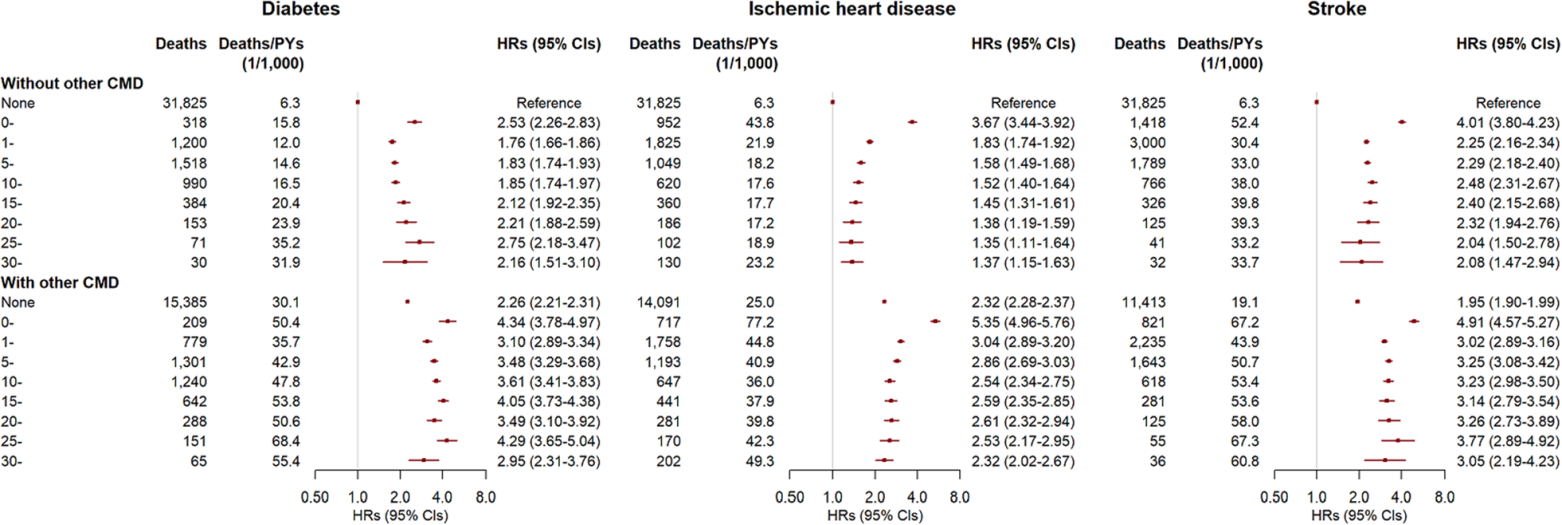
Risks of all-cause mortality by duration of diabetes, ischemic heart disease, and stroke in 512,720 participants. PY, person-year; HR, hazard ratio; CI, confidence interval; CMD, cardiometabolic disease. CMDs included diabetes, ischemic heart disease, and stroke. The status and durations of CMDs were collected at baseline and updated during follow-up. Multivariable models were stratified by age in the 5-year interval and study area, and adjusted for sex, education, household income, marital status, family history of diabetes, heart attack or stroke, smoking, alcohol drinking, dietary habits, physical activity, body mass index, waist circumference, prevalent hypertension, kidney diseases, and rheumatic heart disease

All three CMDs showed a significantly higher risk of mortality in the first year of diagnosis, with HRs (95% CIs) for a single CMD being 2.53 (2.26–2.83) for diabetes, 3.67 (3.44–3.92) for IHD, and 4.01 (3.80–4.23) for stroke. Subsequently, the mortality risk increased with the duration of diabetes. Compared with participants without any CMD, the HRs (95% CIs) were 1.76 (1.66–1.86) for 1–4 years and 2.16 (1.51–3.10) for ≥ 30 years for those with diabetes only (Fig. 2). The mortality risk decreased with the duration of IHD, with HRs (95% CIs) of 1.83 (1.74–1.92) for 1–4 years and 1.37 (1.15–1.63) for ≥ 30 years. The mortality risk for participants with stroke remained above twice that for those without any CMD. When the participants with a single CMD developed other CMDs (i.e., CMM), there was an increase in the association estimates between disease duration and mortality risk. For example, compared with participants without CMD, the HR (95% CI) of mortality associated with diabetes only was 2.21 (1.88–2.59) for 20–24 years, which, however, increased to 3.49 (3.10–3.92) when coexisting with IHD or stroke (either or both).

**Fig. 3:**
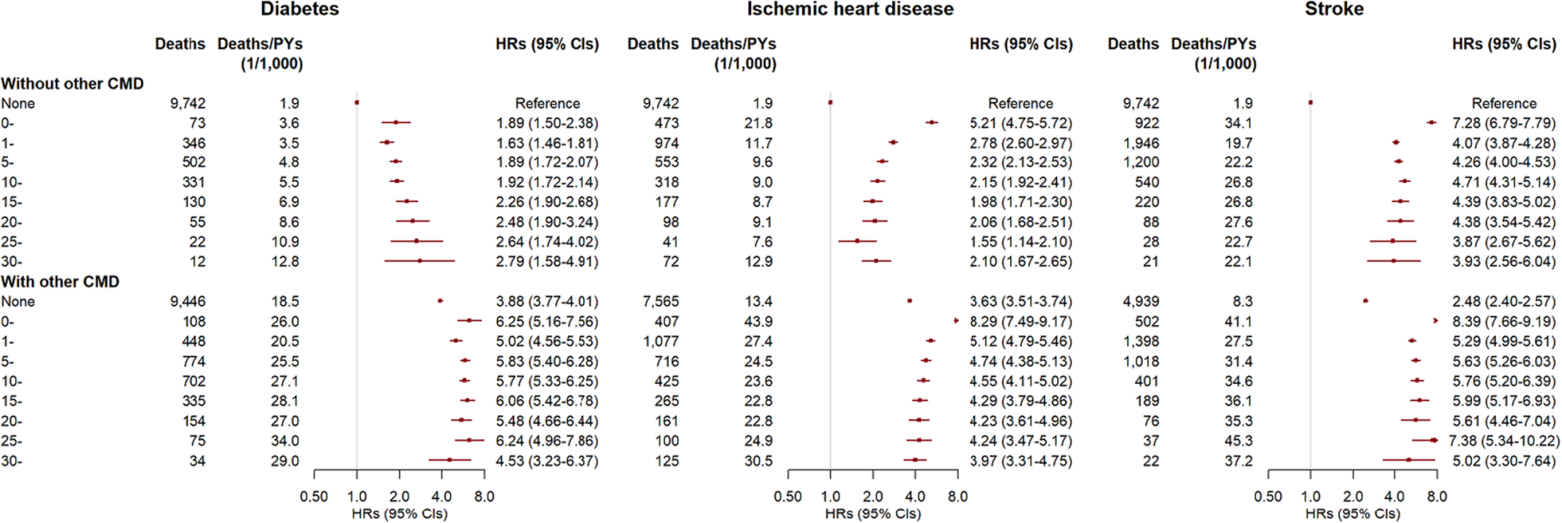
Risks of mortality from circulatory system diseases by duration of diabetes, ischemic heart disease, and stroke in 512,720 participants. PY, person-year; HR, hazard ratio; CI, confidence interval; CMD, cardiometabolic disease. CMDs included diabetes, ischemic heart disease, and stroke. The status and durations of CMDs were collected at baseline and updated during follow-up. Multivariable models were stratified by age in the 5-year interval and study area, and adjusted for sex, education, household income, marital status, family history of diabetes, heart attack or stroke, smoking, alcohol drinking, dietary habits, physical activity, body mass index, waist circumference, prevalent hypertension, kidney diseases, and rheumatic heart disease

The association patterns between the CMD duration and risk of cause-specific mortality were broadly similar to that of all-cause mortality but with different association estimates (Figs. 2 and 3, Additional file 1: Figures S3–S5). One exception was for mortality from cancer. In addition to a significantly higher risk in the first year of diagnosis of any CMD, only patients with diabetes exhibited an increased mortality risk from cancer, which lasted less than 15 years.

### Other sensitivity analyses and stratified analyses

Among 41,029 IS cases and 5155 HS cases occurred during follow-up, 6259 IS cases and 1513 HS cases died afterward, respectively (Additional file 1: Table S1). When the IS and HS were analyzed separately, the association estimates of CMD combinations that include HS were much stronger than that of combinations including IS (Additional file 1: Figure S6). When replacing stroke with IS in the definition of CMDs, the primary analysis results were only slightly attenuated (Additional file 1: Figure S7). In other sensitivity analyses, the results were also not substantially altered (Additional file 1: Figures S8 and S9).

We conducted stratified analyses for associations between the CMD duration and all-cause mortality by sex, residence, and age (Additional file 1: Figures S10–S18). Taking the statistical significance of the test and the actual difference between strata into account, the sex difference in the associations appeared relatively small. In contrast, consistent urban–rural difference and age difference were observed for three CMDs. The associations between the CMD duration and all-cause mortality were stronger among rural than urban participants and among younger (< 60 years) than older participants (≥ 60 years).

## Discussion

In this large prospective cohort of 0.5 million middle-aged and older Chinese adults, three CMDs were associated with higher risk of all-cause and cause-specific mortality, which further increased with the development of CMM. A significantly higher risk of mortality in the first year of diagnosis was observed for all CMDs. Subsequently, the association pattern of disease duration with mortality risk was similar between with or without the presence of CMM but varied among three CMDs. With extended disease duration, the risk of all-cause mortality increased for diabetes, decreased for IHD, and sustained at high level for stroke.

Previous studies found that CMM was associated with an increased risk of mortality [3–13]. The Emerging Risk Factors Collaboration (ERFC) pooled 91 prospective cohorts conducted in North America, Europe, and Australia, involving 689,300 individual data with a median follow-up of 12.8 years. Compared with no diabetes, myocardial infarction (MI), and stroke, the HRs (95% CIs) for diabetes only, MI only, stroke only, concurrent diabetes and MI, concurrent diabetes and stroke, concurrent MI and stroke, and concurrent three CMDs at baseline were 1.8 (1.7–1.9), 2.0 (1.8–2.2), 2.0 (1.9–2.1), 3.6 (3.2–4.1), 3.6 (3.2–4.1), 3.7 (3.2–4.4), 6.0 (5.0–7.1), respectively [6]. Another study was based on the Clinical Practice Research Datalink (CPRD) and followed around 2 million British participants for a median of 7 years. Compared with those without diabetes, IHD, and stroke at baseline, the HRs (95% CIs) for CMD combinations corresponding to the same order as above ERFC were 1.52 (1.51–1.53), 1.51 (1.49–1.52), 1.84 (1.82–1.86), 2.14 (2.11–2.17), 2.53 (2.50–2.57), 2.35 (2.30–2.39), and 3.22 (3.15–3.30), respectively [3].

The association estimates in the ERFC study were higher than that in CKB and CPRD, which could be explained by different study periods. Most cohorts in the ERFC conducted their baseline surveys in the last century and were followed up to the 1990s or mid of 2000s. With improved treatment and management of CMD, it is reasonable for contemporary studies, CKB and CPRD, found lower mortality risks [28–30]. Although our study is comparable with CPRD in both study period and disease definitions, we observed stronger association estimates. It may result from the lower quantity and quality of medical resources in China than the UK [22]. We also observed higher association estimates of each CMD with mortality in the first 20 years after diagnosis in rural than urban populations, supporting above explanation (Additional file 1: Figures S13–S15).

In the present study, there was a particularly high risk for death in the first year of CMD diagnosis, which was consistently observed in previous studies exploring the mortality risk by duration of incident diabetes or IHD [14, 15]. This phenomenon may be attributed to poor short-term prognosis and high death risk associated with acute heart attack and stroke, as well as acute complications of undiagnosed diabetes patients (e.g., diabetic ketoacidosis). For patients who survived the first year of diagnosis, the mortality risks differently changed with duration of diabetes, IHD, and stroke.

In line with previous studies [11, 13, 16–21], we found that the mortality risk increased with duration of diabetes after the first year of diagnosis. We further found that such a trend was mainly due to the contribution of mortality from circulatory system diseases and other causes. In contrast, the increased risk of mortality from cancer attenuated gradually with extended diabetes duration and disappeared after 15 years. A prospective cohort study of over 740 thousand Australian patients with type 2 diabetes also observed the opposite association patterns of disease duration with mortality from cardiovascular diseases versus from cancer [31]. It is possible that diabetes patients with long duration had more frequent contact with the doctors, enabling early detection of cancer and better prognosis. It may also be explained by hyperinsulinemia in early diabetes. With the exhaustion of β cell function, insulin secretion depleted, followed by a decrease in cancer risk [32].

Previous studies observed decreased risk of mortality with duration of IHD [14, 15]. A Danish study compared the mortality risk of 21,693 incident MI cases with that of general population. The HRs (95% CIs) for duration of ≤ 30 days, 31–365 days, 1–10 years, and 11–30 years were 298 (231–385), 7.13 (6.32–8.04), 3.36 (3.20–3.52), and 2.69 (2.59–2.79), respectively [15]. Due to the large sample size and long follow-up periods, the present study observed a clearer time trend for the association and further confirmed that such a decrease was mainly driven by decreased risks of mortality from circulatory and respiratory system diseases.

In our study, the risks of all-cause and cause-specific mortality associated with stroke remained high for a long time, except for cancer mortality. Only one study has examined mortality risk by duration of prevalent stroke in 11,728 older Australian men with a median follow-up of 12.6 years. Compared with no stroke at baseline, they found that the HRs (95% CIs) for duration of < 5, 5–9, 10–14, 15–19, 20–24, 25–29, ≥ 30 years were 1.18 (0.81–1.71), 1.77 (1.48–2.10), 1.62 (1.39–1.89), 1.47 (1.21–1.78), 1.59 (1.24–2.05), 1.01 (0.68–1.50), and 1.26 (0.71–2.23), respectively [18].

When examining the association of CMDs and CMM with mortality, most previous studies were only based on baseline disease status and did not consider the change during follow-up [3, 5–10, 12]. Our sensitivity analysis showed that the definition method of CMD (i.e., incident versus prevalent cases) significantly affected the association estimates. The association estimates of mortality with time-updated disease status during follow-up were stronger than that with baseline disease status for IHD and stroke but lower for diabetes. Previous studies had similar results [4, 13]. One possible explanation was the missed acute cardiovascular cases when only prevalent cases were considered. Also, as observed in our study, the risk of mortality increased with duration of diabetes and decreased with duration of IHD. Our results suggest that incident CMD cases are preferred to the prevalent ones to estimate the risk of mortality. If prevalent cases are inevitably used, findings should be interpreted in the light of duration of CMDs in their population.

The strengths of our study lie in the large sample size and long-term follow-up, resulting in large number of incident CMD cases and deaths. Therefore, a high statistical power enabled us to comprehensively analyze and compare the associations with mortality risk among three CMDs, between a single CMD and CMM, and among different durations of CMDs. Also, the disease and vital status and corresponding dates of diagnosis or death were obtained through the linkage to the established systems, which enabled the time-updated analyses and avoided potential survival bias induced by using only prevalent cases. Finally, the nationwide geographically spread population of CKB, with a broad range of ages and diverse sociodemographic characteristics, enhanced the external validity.

We acknowledge some limitations. First, our study may miss some asymptomatic or mild diabetes because incident cases were primarily identified based on information on hospital admission from the national insurance claim database. Second, CMDs, except for acute MI and stroke, commonly have an insidious onset. Therefore, the diagnosis date recorded in our study may be later than actual onset, like other studies of NCDs. Third, unknown or unmeasured factors may induce residual confounding.

In conclusion, our study confirmed the positive association between the number of CMDs and risks of all-cause and cause-specific mortality. Based on the same population and design, we found that the mortality risk of CMD patients varied by disease, duration, and the presence of CMM. For health professionals, a holistic assessment of these information has the potential to improve disease management. The prevalence of CMDs remains high worldwide. Besides prevention of first CMD, disease management of CMD patients should also be enhanced through lifestyle intervention and effective treatment for prevalent CMD to prevent the occurrence of CMM and reduce the mortality risk of those with CMDs or CMM.

## Supplementary Information

**Additional file 1.** Additional Table S1 and Figures S1–S18.

## Data Availability

Details of how to access China Kadoorie Biobank data and details of the data release schedule are available from www.ckbiobank.org/site/Data+Access.
