# Supplementary material for: Duration-dependent impact of cardiometabolic diseases and multimorbidity on all-cause and cause-specific mortality: a prospective cohort study of 0.5 million participants
Source: Cardiovasc Diabetol. 2023 Jun 12;22:135. doi: 10.1186/s12933-023-01858-9 (PMC10262418; doi:10.1186/s12933-023-01858-9)
Supplement: Supplementary file 1 — Additional file 1. Additional Table S1 and Figures S1–S18. [file 12933_2023_1858_MOESM1_ESM.docx]

Table S1. Number of cases of cardiometabolic diseases at baseline and during follow-up and the corresponding number of deaths.

|  | ICD-10 | Prevalent cases at baseline | |  | Incident cases during follow-up | |  | Cases at baseline and during follow-up | |
| --- | --- | --- | --- | --- | --- | --- | --- | --- | --- |
|  |  | No. of cases | No. of deaths |  | No. of cases | No. of deaths |  | No. of cases | No. of deaths |
| Diabetes | E10-E14 | 30,298 | 7,487 |  | 17,884 | 1,610 |  | 49,362 | 9,339 |
| IHD | I20-I25 | 15,471 | 3,986 |  | 37,096 | 5,104 |  | 58,489 | 10,633 |
| Stroke (total) | I60, I61, I63, I64 | 8,883 | 3,496 |  | 45,265 | 7,422 |  | 63,777 | 13,311 |
| IS | I63 |  |  |  | 41,029 | 6,259 |  |  |  |
| HS | I61 |  |  |  | 5,155 | 1,513 |  |  |  |

IHD, ischemic heart disease; IS, ischemic stroke; HS, hemorrhagic stroke.

Number of incident cases during follow-up was calculated among participants without a prior diagnosis of diabetes, IHD, or stroke at baseline (n=463,178).

The CMD newly documented within 30 days before death during follow-up was not considered an incident case.


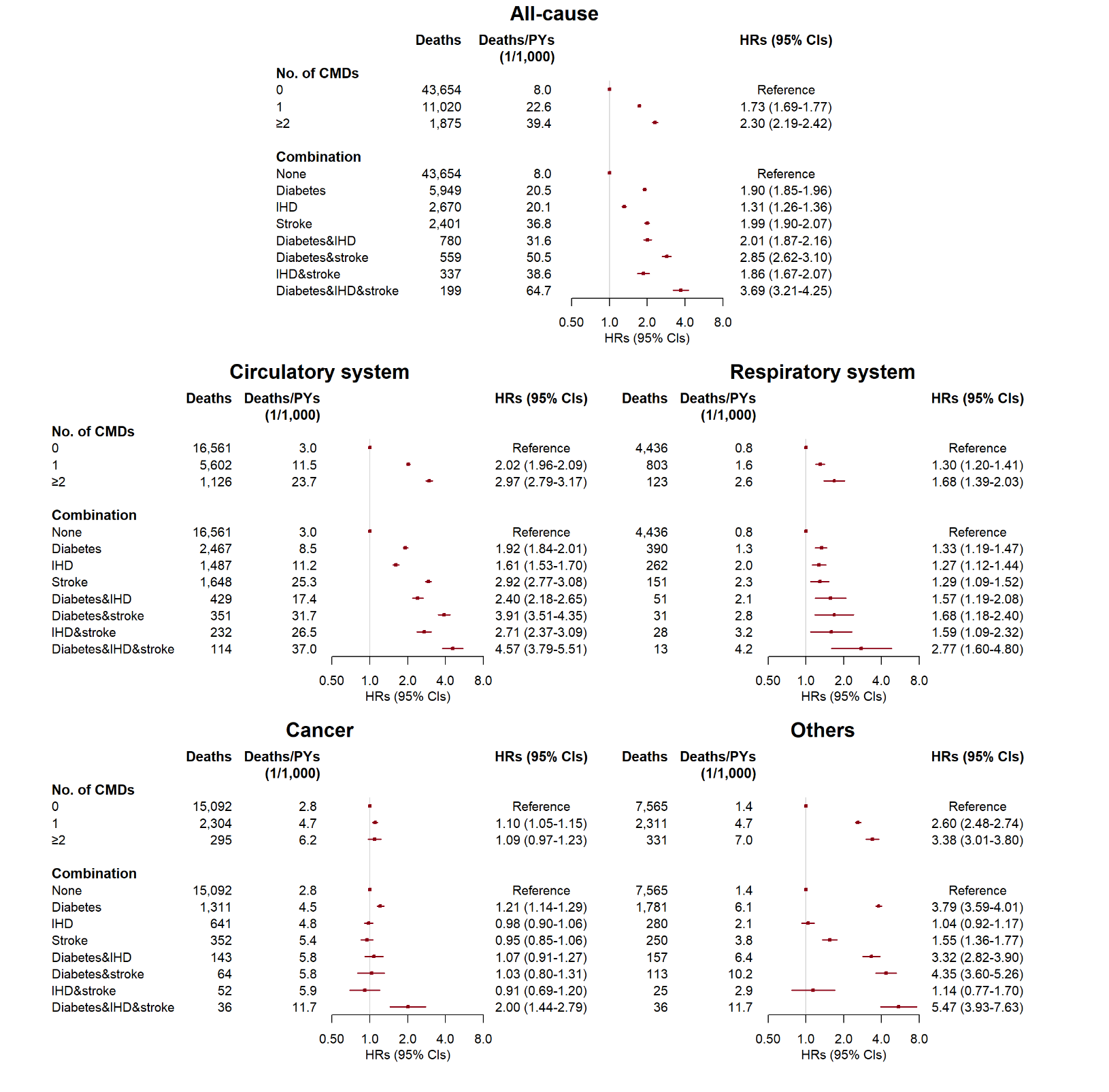
Figure S1. Risks of all-cause and cause-specific mortality by baseline cardiometabolic disease status in 512,720 participants.

PY, person-year; HR, hazard ratio; CI, confidence interval; CMD, cardiometabolic disease; IHD, ischemic heart disease.

CMDs included diabetes, IHD, and stroke. Multivariable models were stratified by age in the five-year interval and study area, and adjusted for sex, education, household income, marital status, family history of diabetes, heart attack or stroke, smoking, alcohol drinking, dietary habits, physical activity, body mass index, waist circumference, prevalent hypertension, kidney diseases, and rheumatic heart disease.


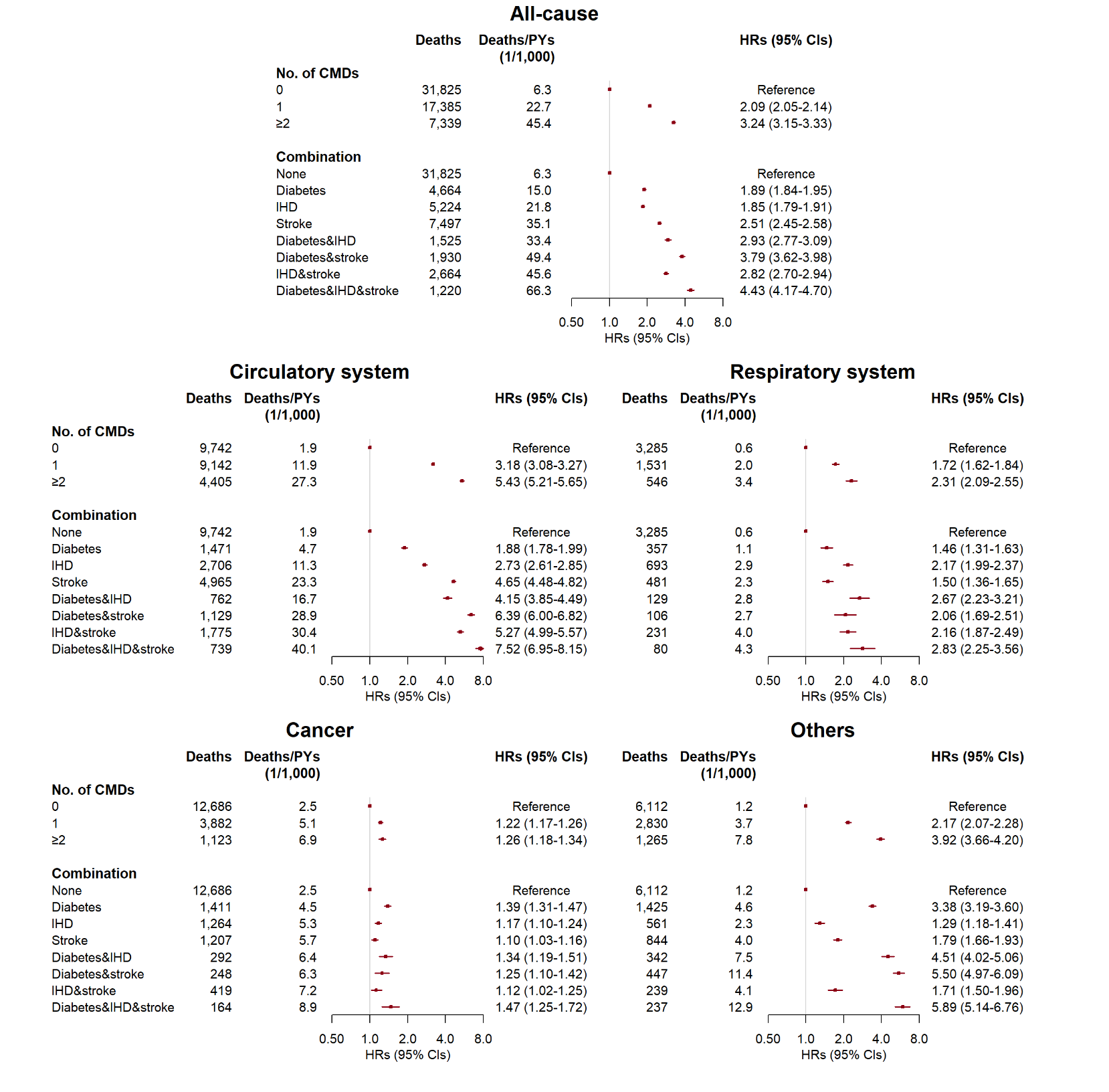
Figure S2. Risks of all-cause and cause-specific mortality by baseline and updated cardiometabolic disease status during follow-up in 512,720 participants.

PY, person-year; HR, hazard ratio; CI, confidence interval; CMD, cardiometabolic disease; IHD, ischemic heart disease.

CMDs included diabetes, IHD, and stroke. Multivariable models were stratified by age in the five-year interval and study area, and adjusted for sex, education, household income, marital status, family history of diabetes, heart attack or stroke, smoking, alcohol drinking, dietary habits, physical activity, body mass index, waist circumference, prevalent hypertension, kidney diseases, and rheumatic heart disease.


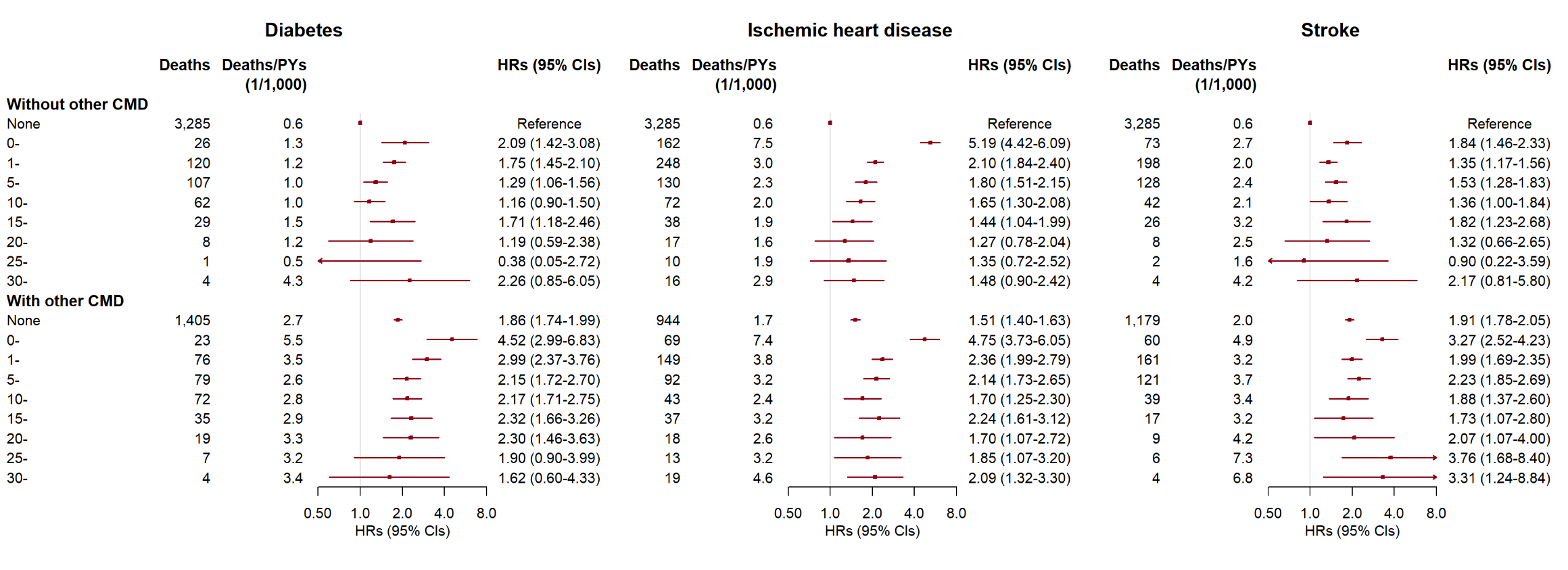
Figure S3. Risks of mortality from respiratory system diseases by duration of diabetes, ischemic heart disease, and stroke in 512,720 participants.

PY, person-year; HR, hazard ratio; CI, confidence interval; CMD, cardiometabolic disease.

CMDs included diabetes, ischemic heart disease, and stroke. The status and durations of CMDs were collected at baseline and updated during follow-up. Multivariable models were stratified by age in the five-year interval and study area, and adjusted for sex, education, household income, marital status, family history of diabetes, heart attack or stroke, smoking, alcohol drinking, dietary habits, physical activity, body mass index, waist circumference, prevalent hypertension, kidney diseases, and rheumatic heart disease.


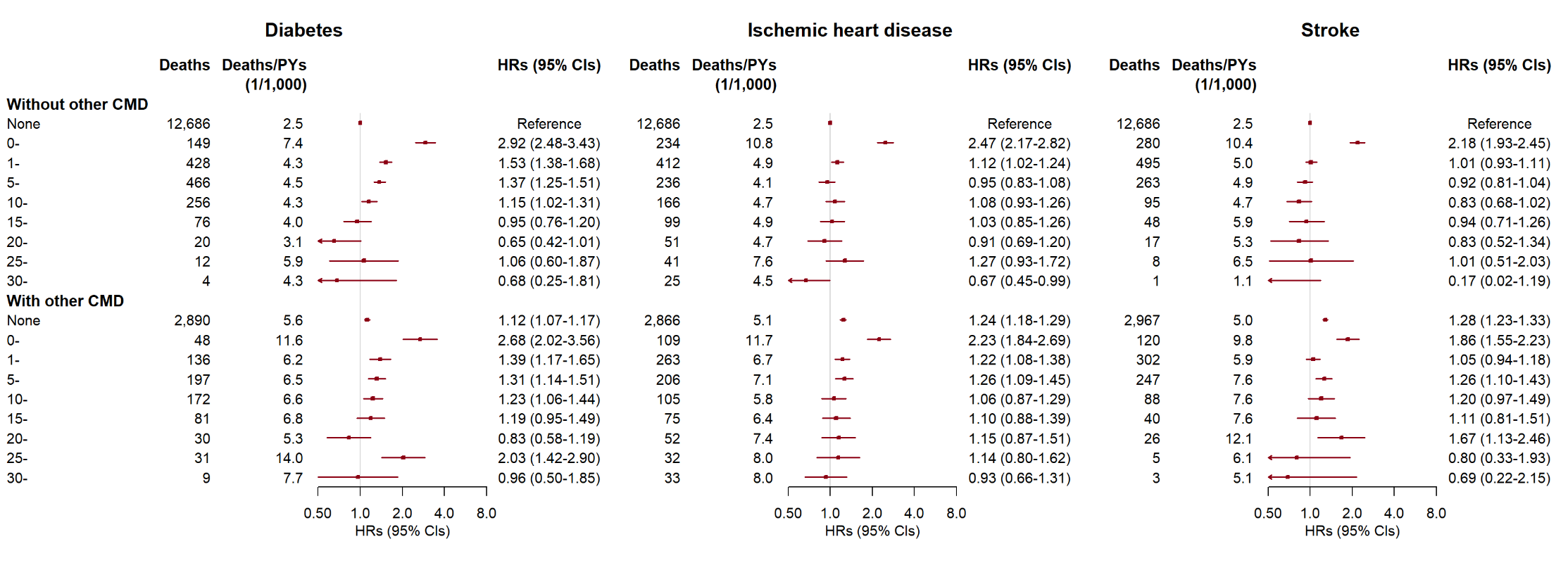
Figure S4. Risks of mortality from cancer by duration of diabetes, ischemic heart disease, and stroke in 512,720 participants.

PY, person-year; HR, hazard ratio; CI, confidence interval; CMD, cardiometabolic disease.

CMDs included diabetes, ischemic heart disease, and stroke. The status and durations of CMDs were collected at baseline and updated during follow-up. Multivariable models were stratified by age in the five-year interval and study area, and adjusted for sex, education, household income, marital status, family history of diabetes, heart attack or stroke, smoking, alcohol drinking, dietary habits, physical activity, body mass index, waist circumference, prevalent hypertension, kidney diseases, and rheumatic heart disease.


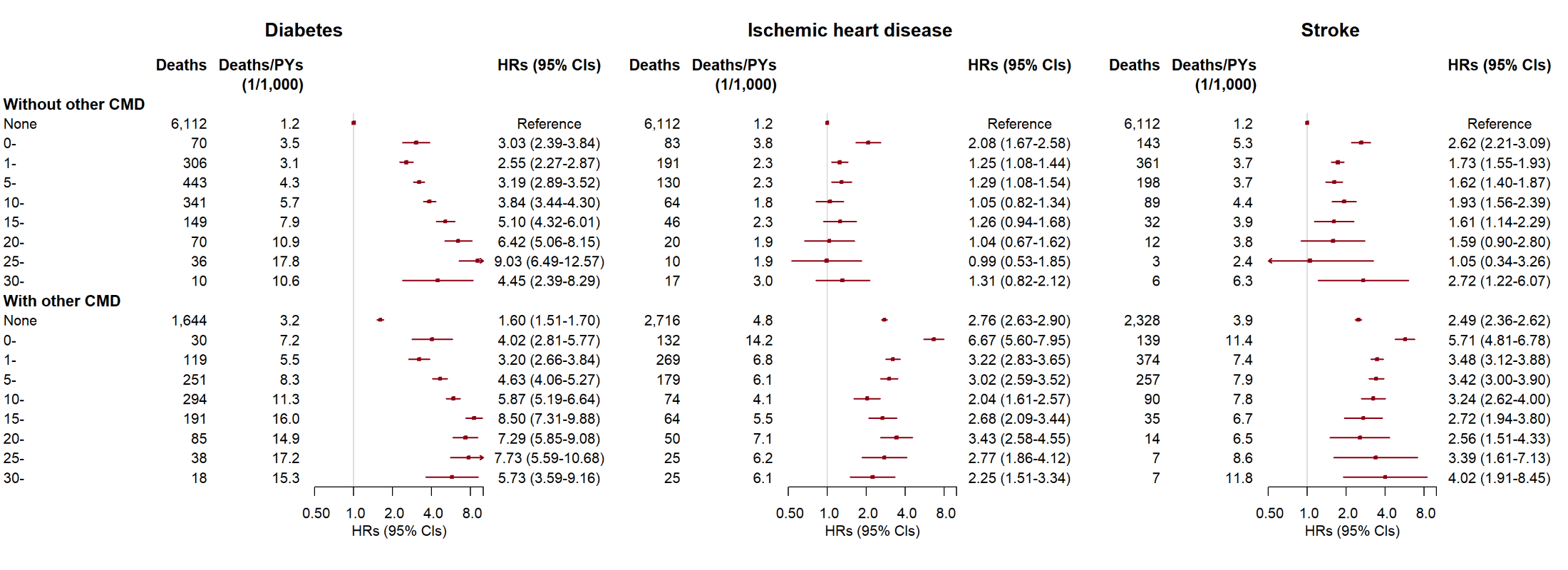
Figure S5. Risks of mortality from other causes by duration of diabetes, ischemic heart disease, and stroke in 512,720 participants.

PY, person-year; HR, hazard ratio; CI, confidence interval; CMD, cardiometabolic disease.

CMDs included diabetes, ischemic heart disease, and stroke. The status and durations of CMDs were collected at baseline and updated during follow-up. Multivariable models were stratified by age in the five-year interval and study area, and adjusted for sex, education, household income, marital status, family history of diabetes, heart attack or stroke, smoking, alcohol drinking, dietary habits, physical activity, body mass index, waist circumference, prevalent hypertension, kidney diseases, and rheumatic heart disease.


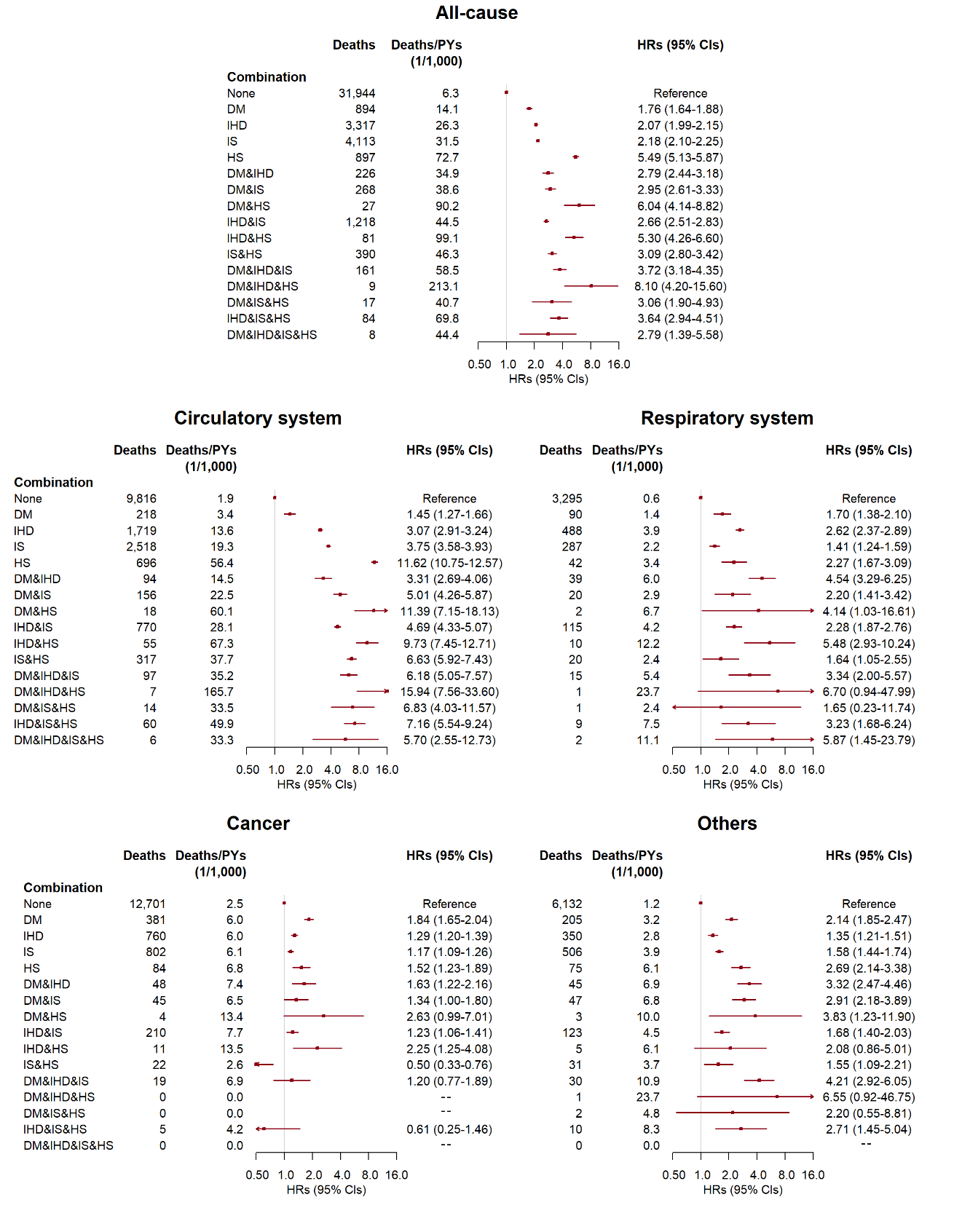
Figure S6. Risks of all-cause and cause-specific mortality by updated status of diabetes, ischemic heart disease, ischemic stroke, and hemorrhagic stroke during follow-up in 463,178 participants.

PY, person-year; HR, hazard ratio; CI, confidence interval; CMD, cardiometabolic disease; DM, diabetes; IHD, ischemic heart disease; IS, ischemic stroke; HS, hemorrhagic stroke.

CMDs included DM, IHD, IS, and HS. Participants with a prior diagnosis of DM, IHD, or stroke at baseline were excluded. Multivariable models were stratified by age in the five-year interval and study area, and adjusted for sex, education, household income, marital status, family history of diabetes, heart attack or stroke, smoking, alcohol drinking, dietary habits, physical activity, body mass index, waist circumference, prevalent hypertension, kidney diseases, and rheumatic heart disease.

Figure S7. Risks of all-cause and cause-specific mortality by updated status of diabetes, ischemic heart disease, and ischemic stroke during follow-up in 463,178 participants.


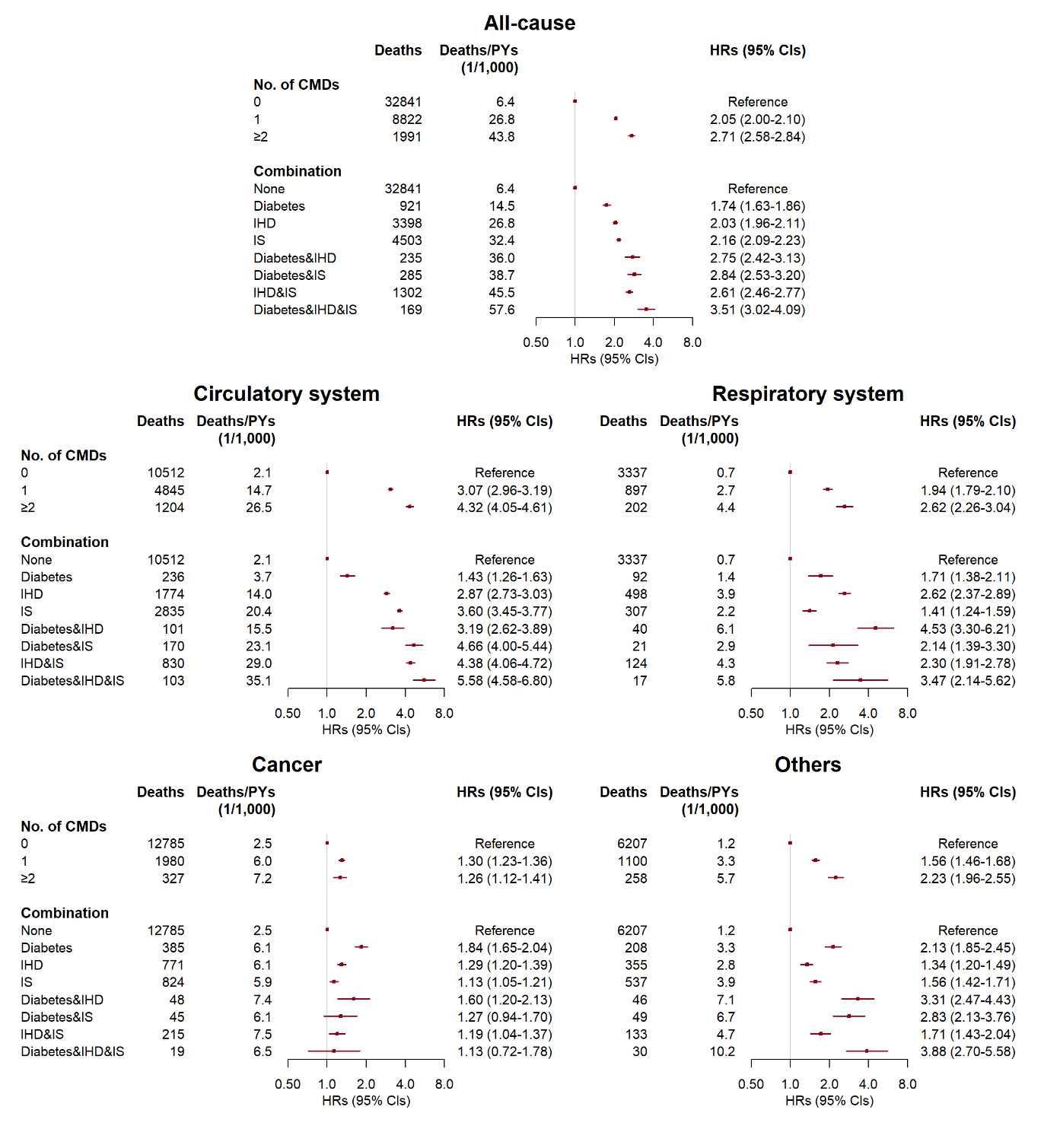


PY, person-year; HR, hazard ratio; CI, confidence interval; CMD, cardiometabolic disease; IHD, ischemic heart disease; IS, ischemic stroke.

CMDs included diabetes, IHD, and IS. Participants with a prior diagnosis of diabetes, IHD, or stroke at baseline were excluded. Multivariable models were stratified by age in the five-year interval and study area, and adjusted for sex, education, household income, marital status, family history of diabetes, heart attack or stroke, smoking, alcohol drinking, dietary habits, physical activity, body mass index, waist circumference, prevalent hypertension, kidney diseases, and rheumatic heart disease.


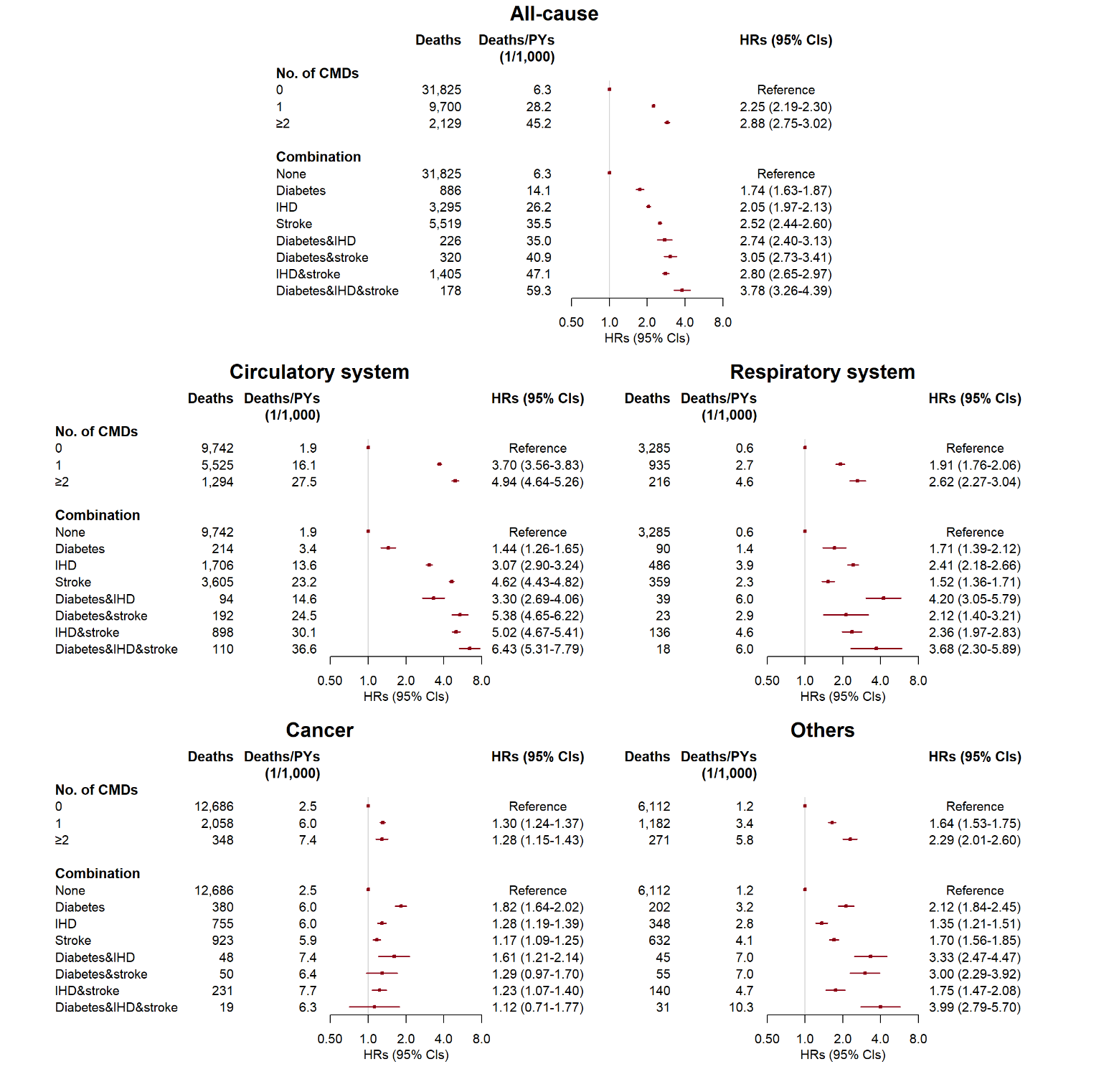
Figure S8. Risks of all-cause and cause-specific mortality by updated cardiometabolic disease status during follow-up after excluding deaths during the first two years of follow-up in 459,195 participants.

PY, person-year; HR, hazard ratio; CI, confidence interval; CMD, cardiometabolic disease; IHD, ischemic heart disease.

CMDs included diabetes, IHD, and stroke. Participants with a prior diagnosis of diabetes, IHD, or stroke at baseline were excluded. Multivariable models were stratified by age in the five-year interval and study area, and adjusted for sex, education, household income, marital status, family history of diabetes, heart attack or stroke, smoking, alcohol drinking, dietary habits, physical activity, body mass index, waist circumference, prevalent hypertension, kidney diseases, and rheumatic heart disease.


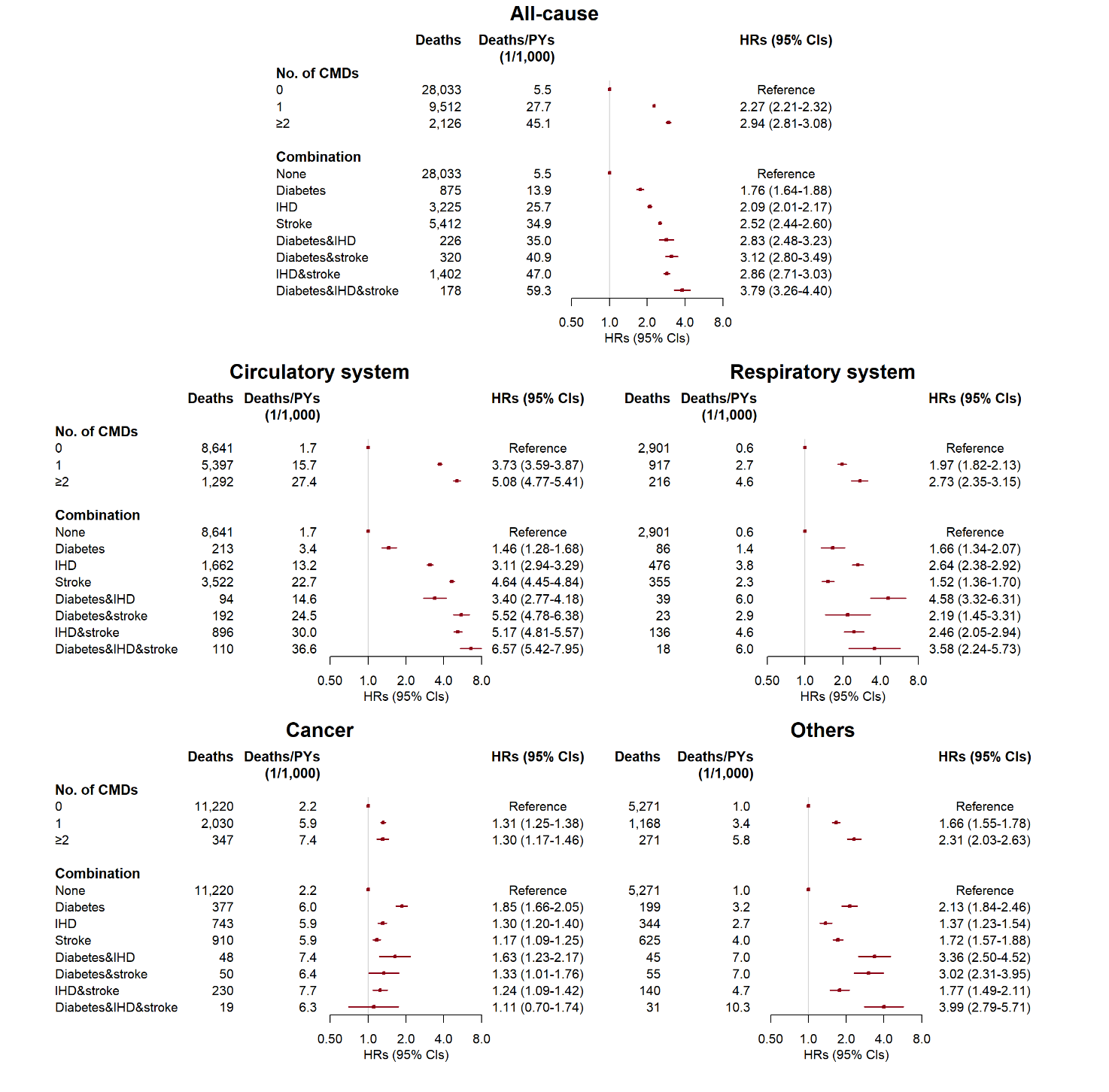
Figure S9. Risks of all-cause and cause-specific mortality by updated cardiometabolic disease status during follow-up after additional adjustments in 463,178 participants.

PY, person-year; HR, hazard ratio; CI, confidence interval; CMD, cardiometabolic disease; IHD, ischemic heart disease.

CMDs included diabetes, IHD, and stroke. Participants with a prior diagnosis of diabetes, IHD, or stroke at baseline were excluded. Multivariable models were stratified by age in the five-year interval and study area, and adjusted for sex, education, household income, marital status, family history of diabetes, heart attack or stroke, smoking, alcohol drinking, dietary habits, physical activity, body mass index, waist circumference, prevalent hypertension, kidney diseases, rheumatic heart disease, cancer, emphysema, and bronchitis, and use of antihypertensive medications, statins, and aspirin.


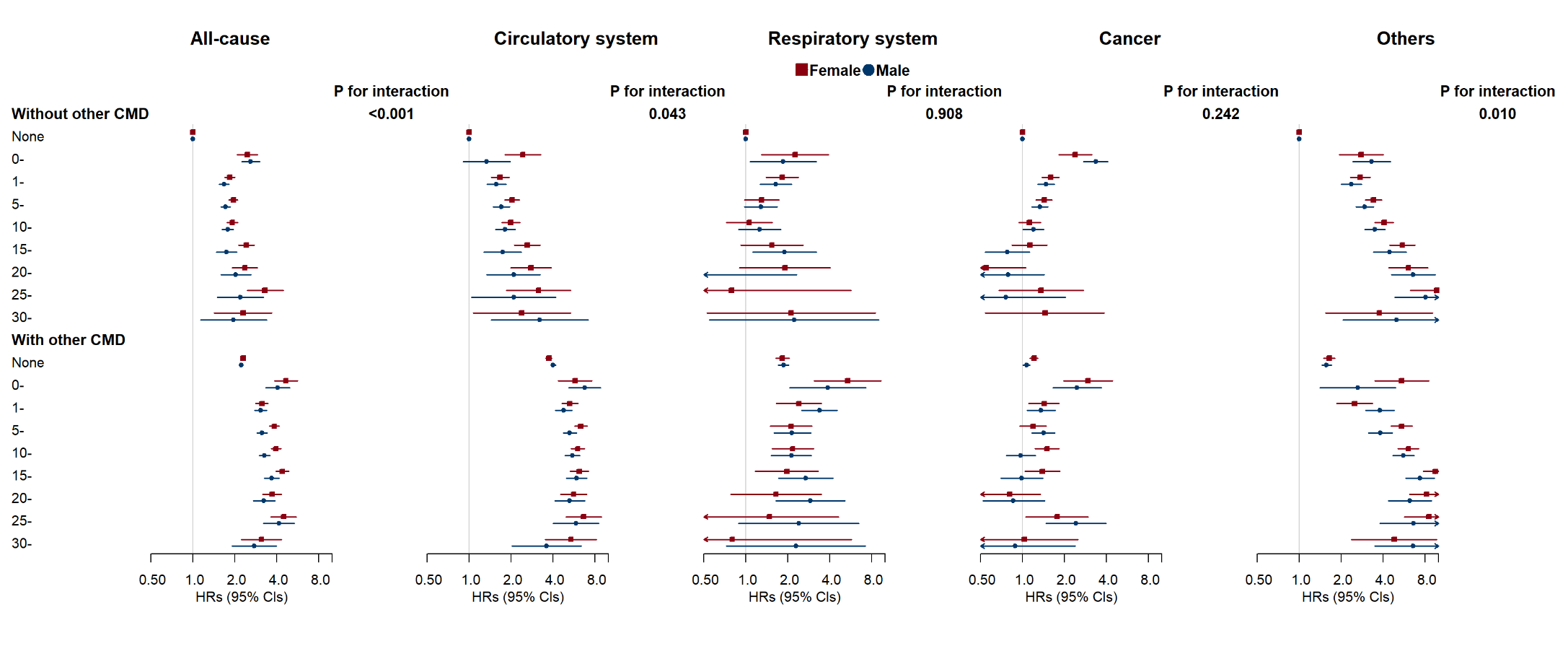
Figure S10. Sex difference in the associations of duration of diabetes with risks of all-cause and cause-specific mortality in 512,720 participants.

HR, hazard ratio; CI, confidence interval; CMD, cardiometabolic disease.

CMDs included diabetes, ischemic heart disease, and stroke. The status and durations of CMDs were collected at baseline and updated during follow-up. Multivariable models were stratified by age in the five-year interval and study area, and adjusted for education, household income, marital status, family history of diabetes, heart attack or stroke, smoking, alcohol drinking, dietary habits, physical activity, body mass index, waist circumference, prevalent hypertension, kidney diseases, and rheumatic heart disease.


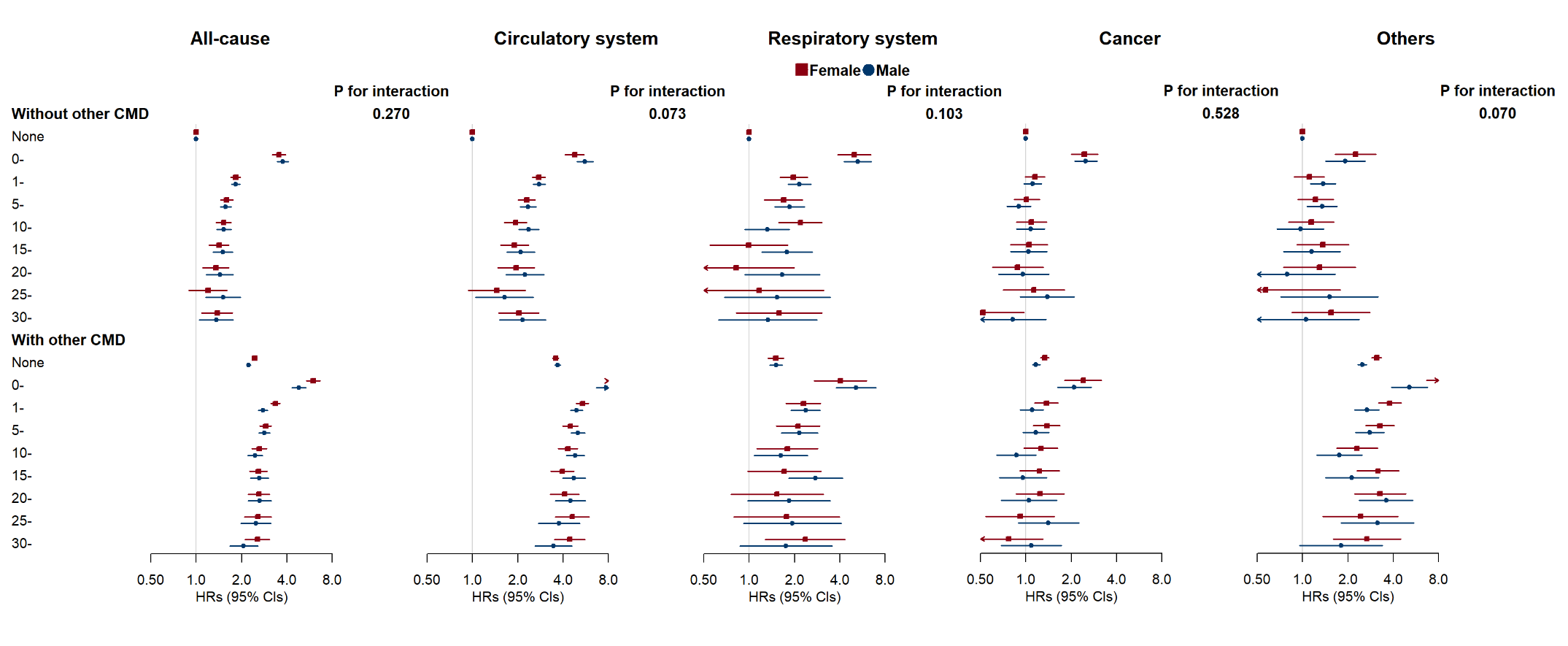
Figure S11. Sex difference in the associations of duration of ischemic heart disease with risks of all-cause and cause-specific mortality in 512,720 participants.

HR, hazard ratio; CI, confidence interval; CMD, cardiometabolic disease.

CMDs included diabetes, ischemic heart disease, and stroke. The status and durations of CMDs were collected at baseline and updated during follow-up. Multivariable models were stratified by age in the five-year interval and study area, and adjusted for education, household income, marital status, family history of diabetes, heart attack or stroke, smoking, alcohol drinking, dietary habits, physical activity, body mass index, waist circumference, prevalent hypertension, kidney diseases, and rheumatic heart disease.


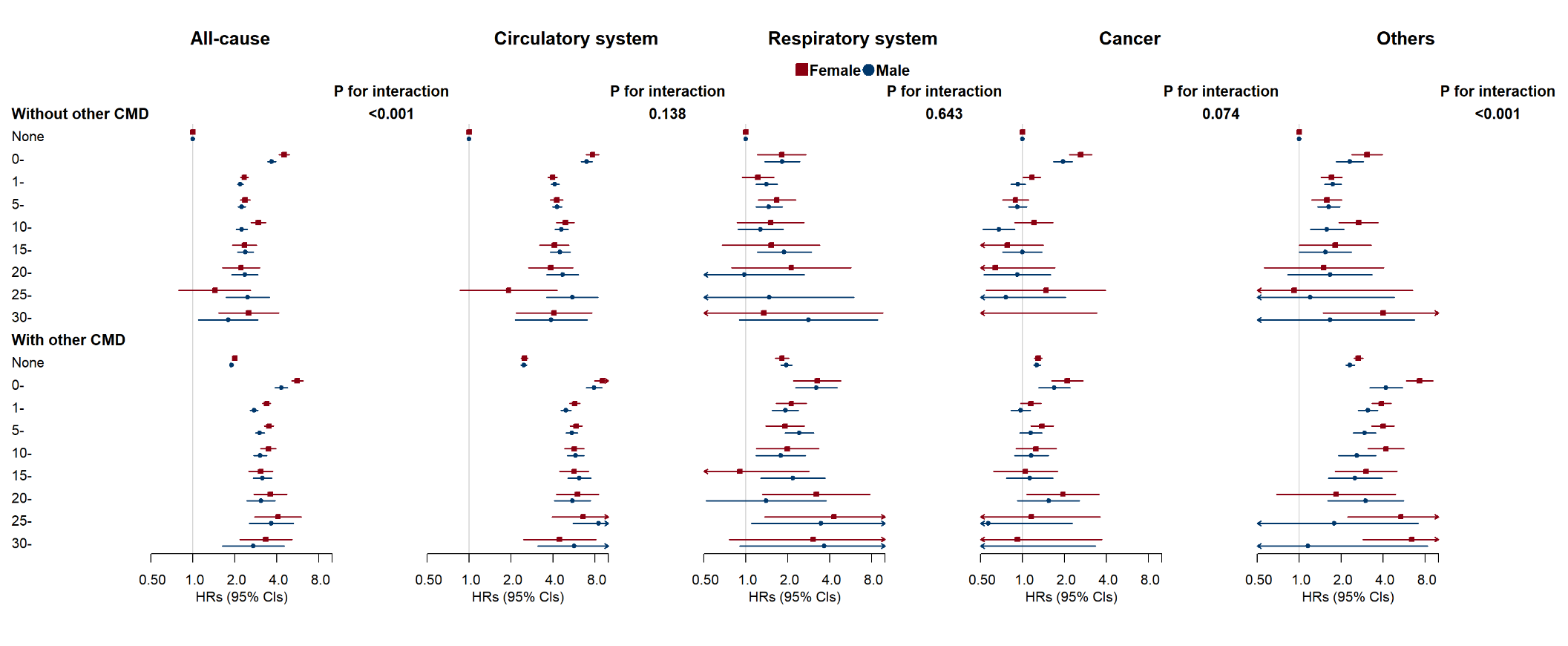
Figure S12. Sex difference in the associations of duration of stroke with risks of all-cause and cause-specific mortality in 512,720 participants.

HR, hazard ratio; CI, confidence interval; CMD, cardiometabolic disease.

CMDs included diabetes, ischemic heart disease, and stroke. The status and durations of CMDs were collected at baseline and updated during follow-up. Multivariable models were stratified by age in the five-year interval and study area, and adjusted for education, household income, marital status, family history of diabetes, heart attack or stroke, smoking, alcohol drinking, dietary habits, physical activity, body mass index, waist circumference, prevalent hypertension, kidney diseases, and rheumatic heart disease.


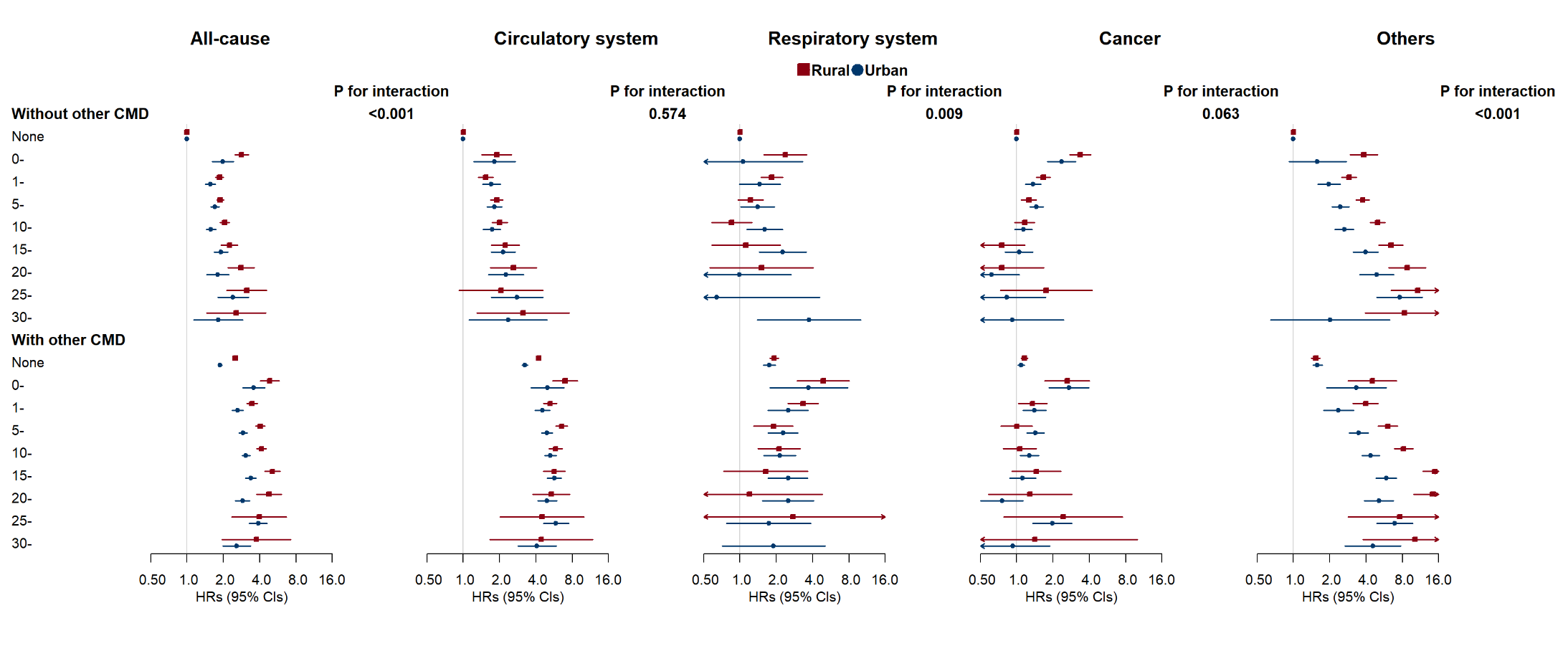
Figure S13. Urban-rural difference in the associations of duration of diabetes with risks of all-cause and cause-specific mortality in 512,720 participants.

HR, hazard ratio; CI, confidence interval; CMD, cardiometabolic disease.

CMDs included diabetes, ischemic heart disease, and stroke. The status and durations of CMDs were collected at baseline and updated during follow-up. Multivariable models were stratified by age in the five-year interval and study area, and adjusted for sex, education, household income, marital status, family history of diabetes, heart attack or stroke, smoking, alcohol drinking, dietary habits, physical activity, body mass index, waist circumference, prevalent hypertension, kidney diseases, and rheumatic heart disease.

Figure S14. Urban-rural difference in the associations of duration of ischemic heart disease with risks of all-cause and cause-specific mortality in 512,720 participants.


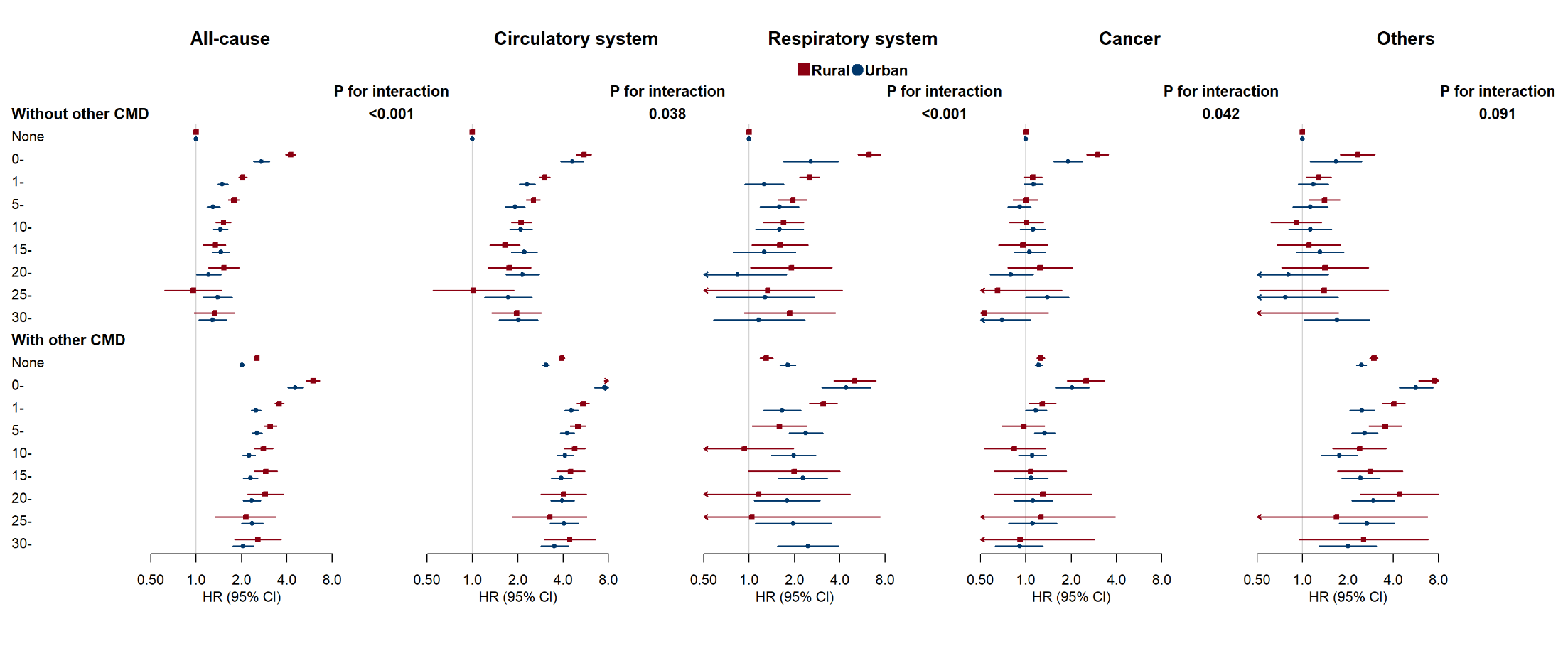
HR, hazard ratio; CI, confidence interval; CMD, cardiometabolic disease.

CMDs included diabetes, ischemic heart disease, and stroke. The status and durations of CMDs were collected at baseline and updated during follow-up. Multivariable models were stratified by age in the five-year interval and study area, and adjusted for sex, education, household income, marital status, family history of diabetes, heart attack or stroke, smoking, alcohol drinking, dietary habits, physical activity, body mass index, waist circumference, prevalent hypertension, kidney diseases, and rheumatic heart disease.


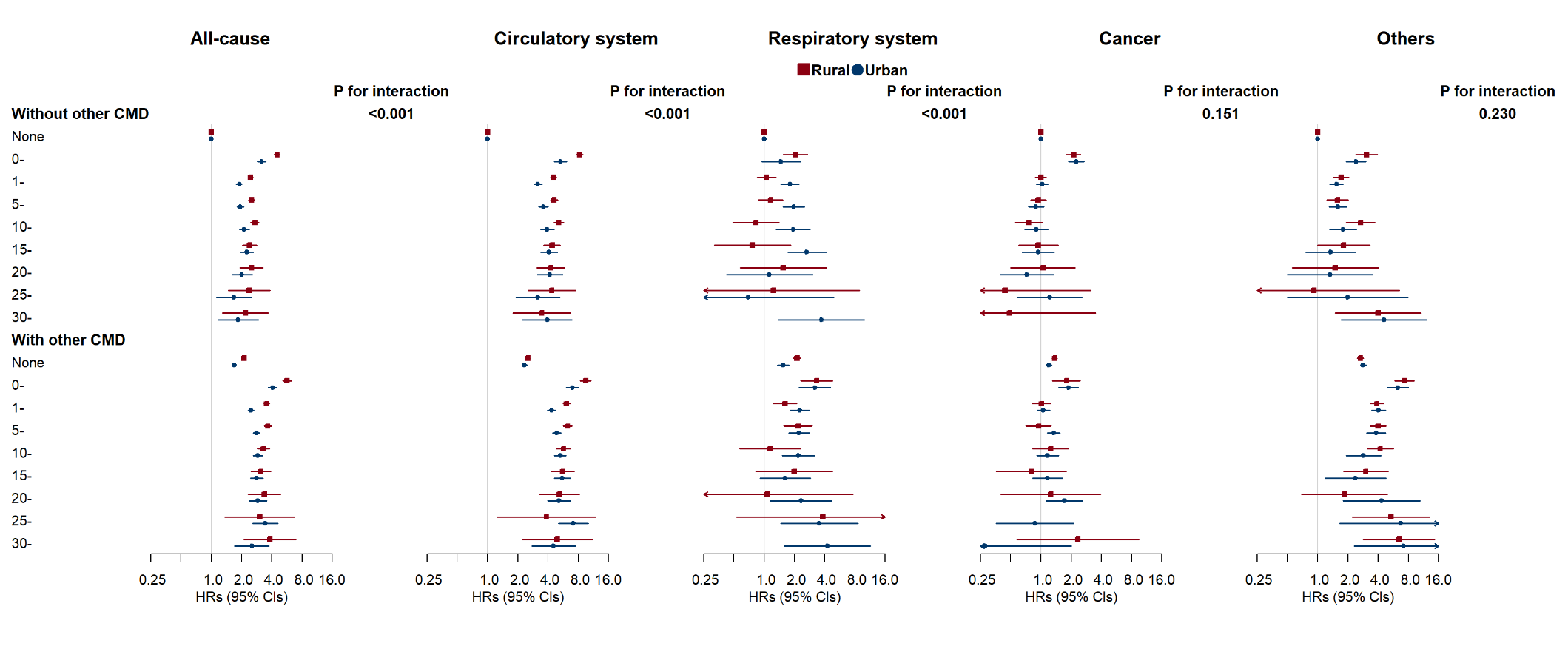
Figure S15. Urban-rural difference in the associations of duration of stroke with risks of all-cause and cause-specific mortality in 512,720 participants.

HR, hazard ratio; CI, confidence interval; CMD, cardiometabolic disease.

CMDs included diabetes, ischemic heart disease, and stroke. The status and durations of CMDs were collected at baseline and updated during follow-up. Multivariable models were stratified by age in the five-year interval and study area, and adjusted for sex, education, household income, marital status, family history of diabetes, heart attack or stroke, smoking, alcohol drinking, dietary habits, physical activity, body mass index, waist circumference, prevalent hypertension, kidney diseases, and rheumatic heart disease.


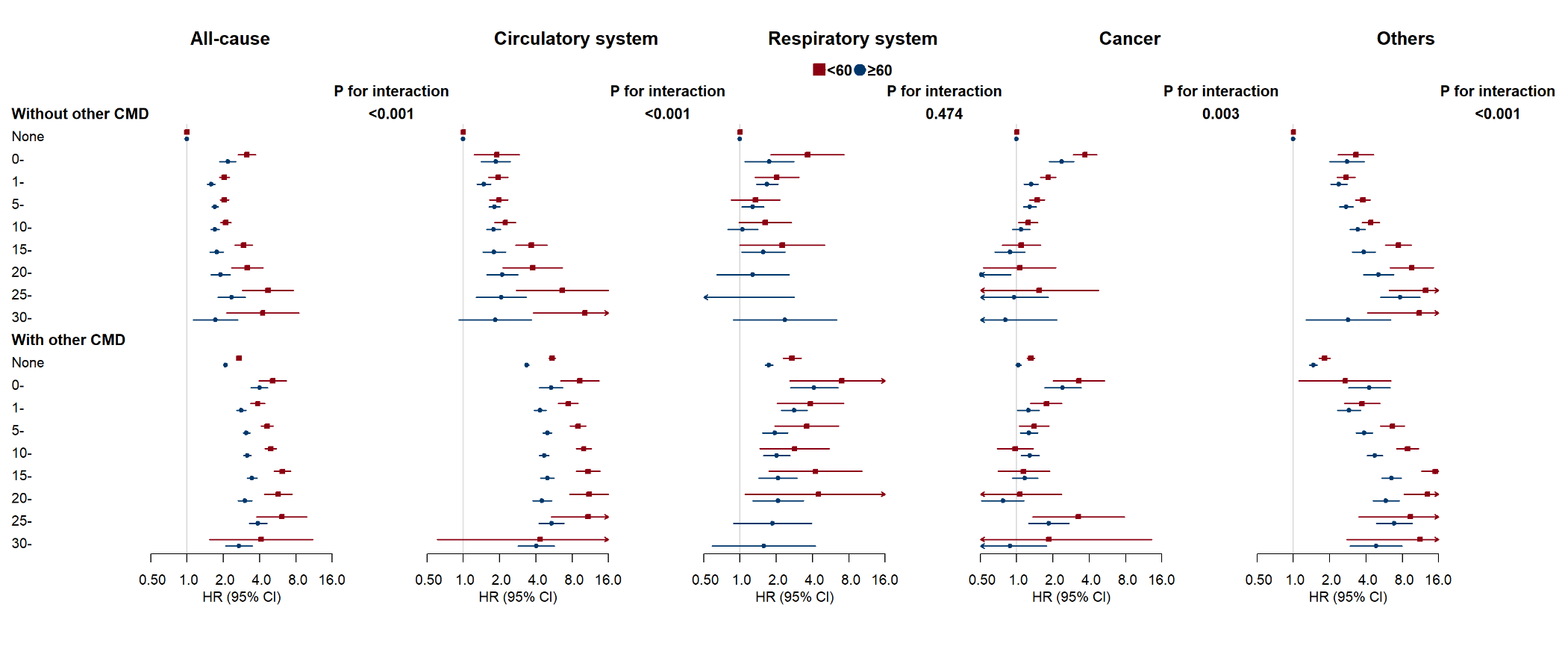
Figure S16. Age difference in the associations of duration of diabetes with risks of all-cause and cause-specific mortality in 512,720 participants.

HR, hazard ratio; CI, confidence interval; CMD, cardiometabolic disease.

CMDs included diabetes, ischemic heart disease, and stroke. The status and durations of CMDs were collected at baseline and updated during follow-up. Multivariable models were stratified by age in the five-year interval and study area, and adjusted for sex, education, household income, marital status, family history of diabetes, heart attack or stroke, smoking, alcohol drinking, dietary habits, physical activity, body mass index, waist circumference, prevalent hypertension, kidney diseases, and rheumatic heart disease.


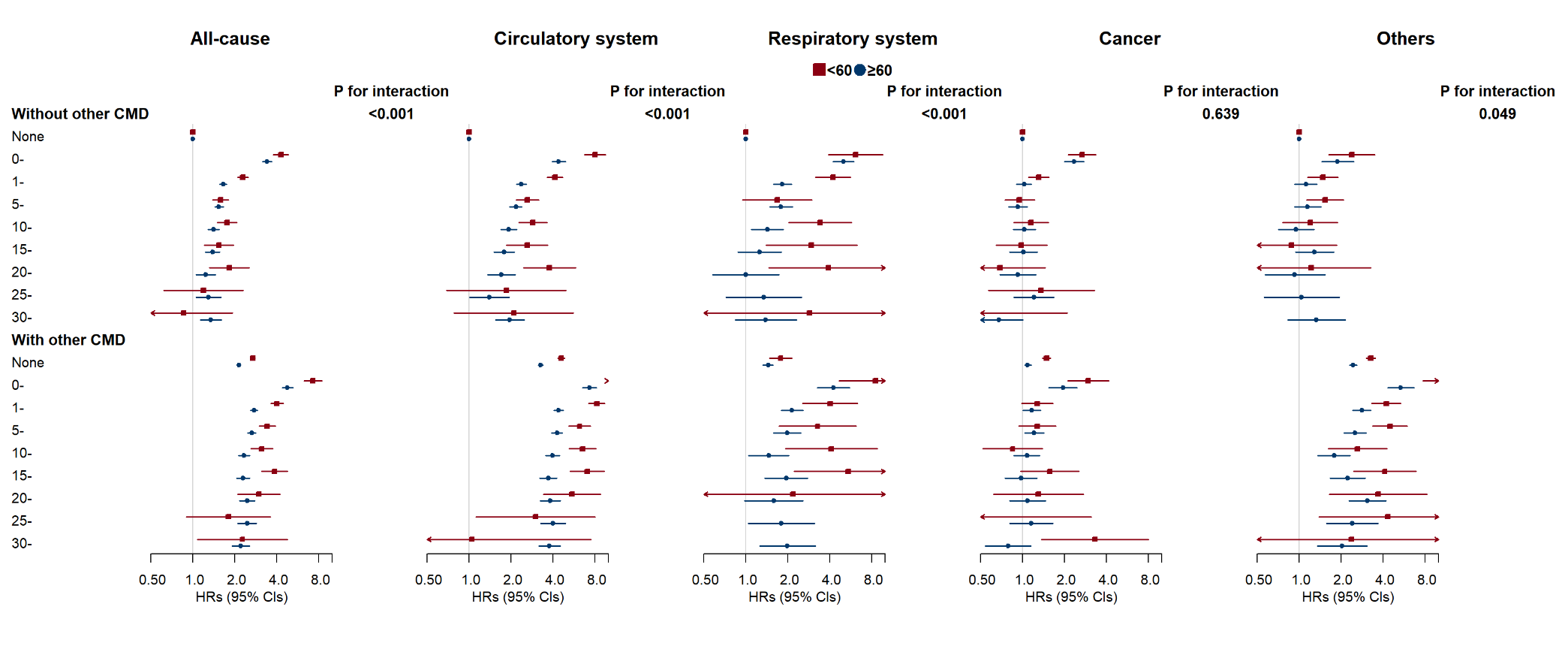
Figure S17. Age difference in the associations of duration of ischemic heart disease with risks of all-cause and cause-specific mortality in 512,720 participants.

HR, hazard ratio; CI, confidence interval; CMD, cardiometabolic disease.

CMDs included diabetes, ischemic heart disease, and stroke. The status and durations of CMDs were collected at baseline and updated during follow-up. Multivariable models were stratified by age in the five-year interval and study area, and adjusted for sex, education, household income, marital status, family history of diabetes, heart attack or stroke, smoking, alcohol drinking, dietary habits, physical activity, body mass index, waist circumference, prevalent hypertension, kidney diseases, and rheumatic heart disease.


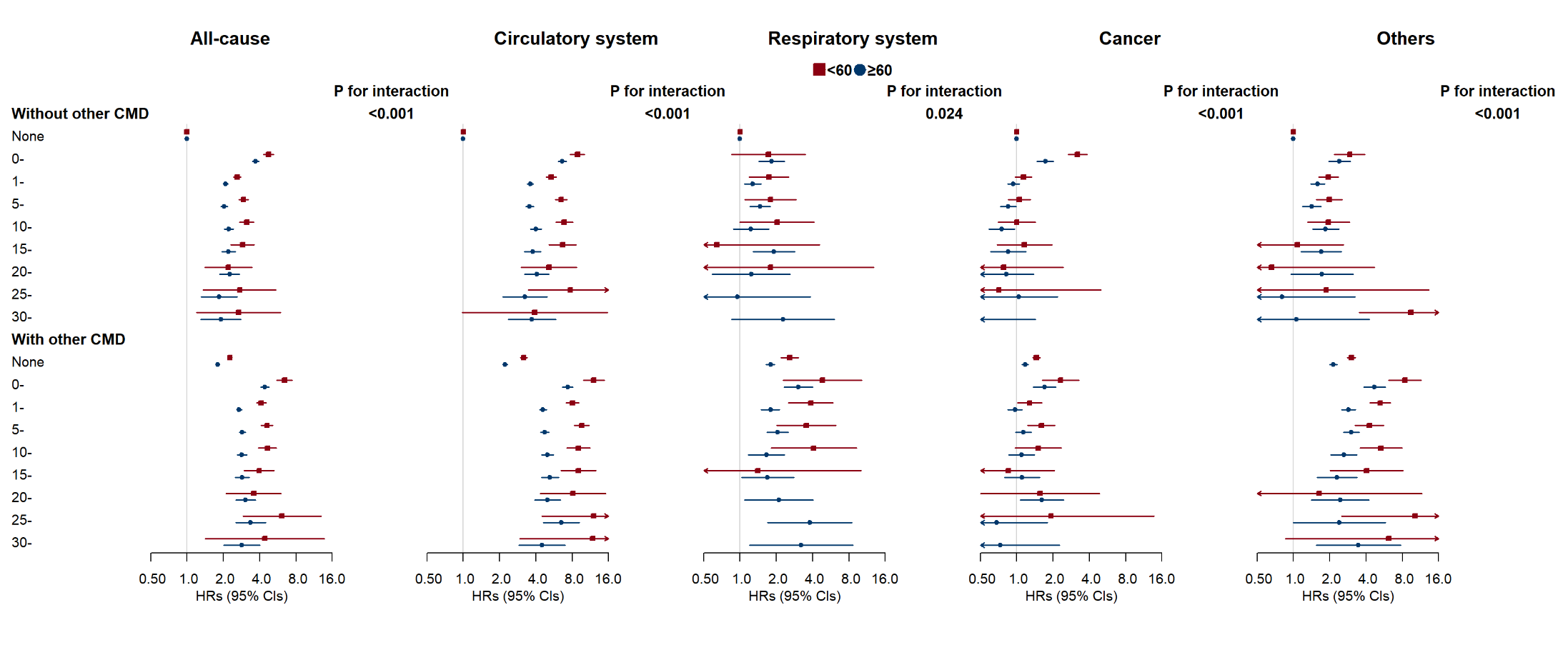
Figure S18. Age difference in the associations of duration of stroke with risks of all-cause and cause-specific mortality in 512,720 participants.

HR, hazard ratio; CI, confidence interval; CMD, cardiometabolic disease.

CMDs included diabetes, ischemic heart disease, and stroke. The status and durations of CMDs were collected at baseline and updated during follow-up. Multivariable models were stratified by age in the five-year interval and study area, and adjusted for sex, education, household income, marital status, family history of diabetes, heart attack or stroke, smoking, alcohol drinking, dietary habits, physical activity, body mass index, waist circumference, prevalent hypertension, kidney diseases, and rheumatic heart disease.
